# 3D Size-Dependent Dynamic Instability Analysis of FG Cylindrical Microshells Subjected to Combinations of Periodic Axial Compression and External Pressure Using a Hermitian *C*^2^ Finite Layer Method Based on the Consistent Couple Stress Theory

**DOI:** 10.3390/ma17040810

**Published:** 2024-02-07

**Authors:** Chih-Ping Wu, Meng-Luen Wu, Hao-Ting Hsu

**Affiliations:** Department of Civil Engineering, National Cheng Kung University, Tainan City 70101, Taiwan

**Keywords:** consistent couple stress theory, dynamic instability, finite layer methods, functionally graded cylindrical shells, static buckling, 3D analysis

## Abstract

This work develops a three-dimensional (3D) weak formulation, based on the consistent couple stress theory (CCST), for analyzing the size-dependent dynamic instability behavior of simply-supported, functionally graded (FG) cylindrical microshells that are subjected to combinations of periodic axial compression and external pressure. In our formulation, the microshells are artificially divided into *n_l_* layers. The displacement components of each individual layer are selected as the primary variables, which are expanded as a double Fourier series in the in-plane domain and are interpolated with Hermitian *C*^2^ polynomials in the thickness direction. Incorporating the layer-wise displacement models into our weak formulation, we develop a Hermitian *C*^2^ finite layer method (FLM) for addressing the current issue. The accuracy and the convergence rate of our Hermitian *C*^2^ FLM are validated by comparing the solutions it produces with the accurate two-dimensional solutions of critical loads and critical pressures of FG cylindrical macroshells and single-walled carbon nanotubes, which were reported in the literature. The numerical results show the effects of the material length-scale parameter, the inhomogeneity index, the radius-to-thickness and length-to-radius ratios, the load magnitude ratio, and the static and dynamic load factors on the first principal and first secondary instability regions of parametric resonance of simply-supported FG cylindrical microshells are significant.

## 1. Introduction

Recently, developing a novel computational method for analyzing various mechanical behaviors of functionally graded (FG) microscale structures has attracted considerable attention. On the one hand, due to the advancement of material technology, FG materials have gradually replaced traditional laminated composite materials (LCMs) in practical application to eliminate their shortcomings, such as delamination, which often occurs at the interfaces between adjacent layers, and stress concentration, which often occurs at the regions where the structure’s dimension abruptly changes [1,2,3]. Furthermore, because FG materials are composed of two-phase or multi-phase materials, which are mixed with specific volume fractions of the constituent materials gradually and smoothly varying in the physical domain of the structure of interest, they can not only remove the above weaknesses of LCMs, but also intrinsically form unique properties of the structures of interest, such as a high stiffness-to-mass ratio, a high strength-to-mass ratio, an excellent corrosion resistance capacity, and an outstanding thermal resistant capacity [4,5]. On the other hand, as manufacturing technology advanced, smaller FG structures could be made. As a result, their scale has progressively reduced from the macro scale to the micron scale, and even to the nanoscale. For example, these miniaturized structural systems include micro-electro-mechanical systems, nano-electro-mechanical systems, micro-sized sensors and actuators, optoelectric and thermoelectric devices, and atomic force microscopes [6,7,8]. As the scale of structures gradually shrinks to the micron scale and the nanoscale, the analytical and numerical methods based on the classical continuum mechanics (CCM) for analyzing FG macroscale structures are no longer applicable due to the manifestation of size-dependent effects. Thus, developing a non-CCM-based theory appropriate for analyzing FG microscale and even nanoscale structures has become essential for academic research [9,10,11].

Yang et al. [12] and Hadjesfandiari and Dargush [13,14] reformulated the original couple stress theory (OCST) [15,16,17] to develop the modified couple stress theory (MCST) and the consistent couple stress theory (CCST), respectively, by requiring the symmetric and skew-symmetric properties of the couple-tress tensor. A benefit of the MCST and the CCST is that instead of two material length-scale coefficients being required to analyze elastic isotropic solids, as in the OCST, only one material length-scale coefficient is required. Furthermore, they successfully applied their models to study the torsion behavior of a thin cylinder and the pure bending behavior of a flat plate with an infinite width.

Cylindrical shells are commonly used in cutting-edge technologies such as aerospace, submarine manufacturing, and nuclear engineering. When such structural components are subjected to periodic loads, the dynamic instability phenomenon will occur in them due to parametric resonance, leading to the amplitude of the transverse vibration of these structural components increasing until they collapse. This dynamic instability phenomenon happens in cylindrical macro- and microshells, which is worth advanced study. Before we address the above issue, a comprehensive survey focusing on the articles that examine the dynamic instability behavior of LCM/FG cylindrical macro- and microshells using various analytical and numerical methods is necessary, and presented in the paragraph below.

Some articles have presented the results of the dynamic instability analysis of laminated cylindrical macroshells. Based on Love’s classical shell theory (LCST), Argento and Scott [18,19] analyzed the dynamic instability behavior of laminated anisotropic circular cylindrical shells subjected to periodic axial compression. In their formulation, the shell’s response was divided into a pre-instability state and a subsequent incremental perturbation state, which could be dynamically unstable. The system equations for the perturbed state are a system of Mathieu–Hill equations [20]. As a result, Bolotin’s method [21] was used to obtain the principal instability regions of parametric resonance of the shells of interest. Based on the first-order shear deformation theory (FSDT), Ganapathi and Balamurugan [22] and Ganapathi and Patel [23] developed a two-node Lagrangian *C*^0^ axisymmetric shell element method to investigate the dynamic instability responses of laminated composite cylindrical and conical shells, respectively, which were subjected to combinations of periodic axial compression and external pressure. Based on Donnell’s classical shell theory (DCST), Sofiyev and Pancar [24] studied the dynamic instability behavior of orthotropic conical shells, which were subjected to periodic axial compression, for which the effect of heterogeneity on the principal instability regions of parametric resonance was examined. In conjunction with the LCST and the differential quadrature (DQ) method, Bert and Birman [25] and Ng et al. [26] examined the dynamic instability response of homogeneous isotropic cylindrical shells and conical shells, respectively, which were subjected to periodic axial compression. In their analyses, the effects of the transverse shear deformation, rotary inertia, and bending deformation in the pre-perturbed state were ignored, and a linear buckling theory was used to determine the principal instability regions of parametric resonance. Finally, based on three-dimensional (3D) thermo-elasticity, Wu and Chiu [27] investigated the thermally induced dynamic instability behavior of laminated composite conical shells subjected to periodic thermal loads with a perturbation method.

Some articles have studied the dynamic instability behavior of FG cylindrical and conical macroshells. Based on a modified DCST, Sofiyev [28] studied the dynamic instability behavior of FG cylindrical sandwich shells subjected to periodic axial compression. Material properties of these shells were assumed to obey an exponential function varying in the thickness direction. The effect of shear deformations on the principal regions of parametric resonance of the sandwich shells was discussed. In another study, Sofiyev [29] combined the FSDT with Galerkin’s method to investigate the dynamic instability behavior of FG conical shells subjected to periodic external pressure. The impacts of some essential factors on the principal instability regions of the excitation frequency were measured and discussed. The factors considered included the shear deformations, the inhomogeneity index, the static and dynamic load factors, and the cone angles. Ganapathi et al. [30] developed a two-node Lagrangian *C*^0^ finite shell element method for the dynamic instability analysis of truncated conical shells by incorporating the FSDT displacement model into the strain energy function of the shells. Pradyumna and Bandyopadhyay [31] investigated the dynamic instability behavior of FG shallow shells subjected to periodic in-plane mechanical loads and thermal loads, where five forms of shallow shells were taken into consideration, including FG cylindrical panels, FG doubly curved shells, FG hyperbolic paraboloid shells, FG doubly curved hypar shells, and FG doubly curved conoid shells.

Some articles examining the dynamic instability behavior of FG cylindrical and conical microshells using two-dimensional (2D) size-dependent shear deformation theories have also been presented. For example, by incorporating the FSDT displacement model into the MCST, Gholami et al. [32] investigated the dynamic instability behavior of FG cylindrical microshells subjected to periodic axial compression. They indicated that the size-dependent effect on the principal instability regions of parametric resonance is significant. Sahmani et al. [33] developed an MCST-based higher-order shear deformation theory (HSDT) to study the dynamic instability behavior of FG cylindrical microshells. They concluded that the bandwidth of the instability region increased when the material length-scale parameter became greater. Finally, Pham and Nguyen [34] developed an MCST-based four-variable refined plate theory to determine the principal instability regions of parametric resonance of FG porous cylindrical microshells. The impacts of some essential factors on the principal instability regions of parametric resonance were discussed, including porosity coefficients, porosity distributions, the static and dynamic load factors, the material length-scale parameter, and the inhomogeneity index.

As mentioned above, almost all analyses for the dynamic instability behavior of FG cylindrical microshells reported in the literature used 2D size-dependent shear deformation cylindrical shell theories based on the MCST. In contrast, the 3D size-dependent elasticity theory based on the CCST was seldom used. Thus, 3D effects, including the thickness stretching effect, the zig-zag deformation effect for laminated cylindrical microshells, and the 3D couple-stress tensor effect, on the instability regions of the excited frequency applied to the microshells are worthy of further investigation.

In recent work, Wu and Hsu [35] and Wu and Lu [36] derived a 3D size-dependent weak formulation based on the CCST, which was used to develop the Lagrangian *C*^0^ and the Hermitian *C*^1^ finite layer methods (FLMs) for analyzing the static bending and free vibration behaviors of FG elastic and piezoelectric microplates, for which the symbol *C^n^* indicates each primary variable was interpolated to satisfy the continuity conditions of its *n*^th^-order derivatives at the interfaces between adjacent nodal surfaces. The numerical results revealed that the convergence rate of the Hermitian *C*^1^ FLM is more rapid than that of the Lagrangian *C*^0^ FLM, and their convergent solutions were close to each other and were in excellent agreement with the exact 3D solutions for macroplates, which were reported in the literature.

To extend the application region of these CCST-based FLMs from the analysis of the mechanical behavior of microplates to that of the mechanical behavior of cylindrical microshells, and also to speed up their convergence rates, in this study, within the framework of the CCST, we aim to develop the Hermitian *C*^2^ FLM to analyze the 3D size-dependent dynamic instability behavior of simply-supported FG cylindrical microshells subjected to combinations of periodic axial compression and periodic external pressure. In our formulation, the instability behavior of the shells of interest is divided into two states, namely the pre-instability state and the subsequent incremental perturbation state. Following the 3D elasticity theory, Soldatos and Ye [37] and Ye and Soldatos [38] determined a set of normal stresses that occurred at the pre-instability state with the state space method, and were regarded as the initial stresses. Afterward, with the developed CCST-based Hermitian *C*^2^ FLM, initial stresses are introduced to the incremental perturbation state to find the principal instability regions of parametric resonance of FG cylindrical microshells.

## 2. The Initial Stresses Induced at the Pre-Instability State

**Figure 1:**
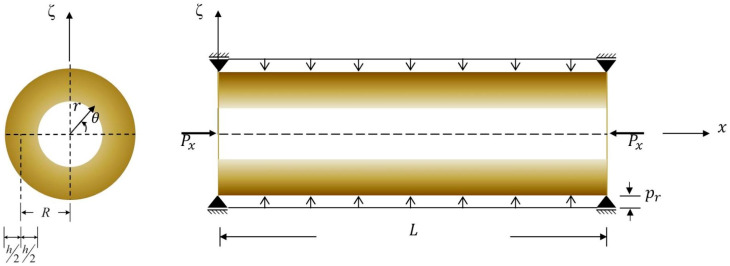
The schematic diagram of a simply supported FG cylindrical microshell subjected to combinations of axial compression.

This work presents the 3D analysis of size-dependent dynamic instability behavior of simply-supported FG cylindrical microshells subjected to combinations of periodic axial compression and external pressure, as shown in Figure 1. Our formulation considers a typical FG cylindrical microshell of thickness *h*, length *L*, and mid-surface radius *R*. The microshell is artificially divided into *n_l_* layers, with a thickness of *h_m_* for the *m*th layer, such that $\sum_{m=1}^{n_{l}}h_{m}=\;h.$ A global cylindrical coordinate system (i.e., *x*, θ, and *r* coordinates) is positioned with its origin at the center of the microshell. A global thickness coordinate ζ is located on the mid-surface of the microshell, and a set of local thickness coordinates $z_{m}\;(m=1,\;\;2,\;\;3,\ldots ,\;\;n_{l})$ is located on the mid-surface of each layer. The relationship between the radial coordinate (*r*) and the global thickness coordinate (ζ) is r=R+ζ. The relationship between the global and local thickness coordinates of the *m*th layer is $\zeta ={\bar{\zeta }}_{m}+z_{m}$, for which ${\bar{\zeta }}_{m}=\left(\zeta_{m}+\zeta_{m-1}\right)/2$ and $\zeta_{m}\;\;\mathrm{and}\;\;\zeta_{m-1}$ are defined as the global thickness coordinates measured from the mid-plane of the microshell to the top and the bottom surfaces of the *m*th layer, respectively.

In Leissa’s linear instability theory [39], in-surface and out-of-surface normal stresses exist in the cylindrical microshell of interest just before instability occurs. The displacement components of the *m*th layer at the pre-buckling state can be obtained by following Soldatos and Ye’s [37] and Ye and Soldatos’s [38] displacement fields, and are given by

(1a) $${\bar{u}}_{x}^{\left(m\right)}=A_{0}\;x,$$

(1b) $${\bar{u}}_{\theta }^{\left(m\right)}=0,$$

and

(1c) $${\bar{u}}_{r}^{\left(m\right)}=\;{\bar{W}}_{0}^{\left(m\right)}\left(\zeta \right)\;m=1,\;2,\cdots ,\;n_{l},$$

where in this section, a bar above a variable represents measurement at the pre-instability state. The symbol $A_{0}$ is an arbitrary constant representing a uniform axial strain induced in the microshell when subjected to combinations of periodic axial compression and external pressure, and its value will be determined later in this article by satisfying the force equilibrium equation in the axial direction at the edges.

Substituting the initial displacement model given in Equations (1a)–(1c) into the basic equations of 3D elasticity theory leads to the normal and shear strain components (${\bar{\varepsilon }}_{\;kk}^{\left(m\right)}\;\;\mathrm{and}\;\;{\bar{\gamma }}_{\;ij}^{\left(m\right)}$) and the normal and shear stress components as follows:

(2a) $${\bar{\varepsilon }}_{\;xx}^{\left(m\right)}=A_{0},$$

(2b) $${\bar{\varepsilon }}_{\;\;\theta \theta }^{\left(m\right)}={\bar{W}}_{\;0}^{\left(m\right)}/r,$$

(2c) $${\bar{\varepsilon }}_{\;\;rr}^{\left(m\right)}={\bar{W}}_{\;0}^{\left(m\right)}{,}_{\zeta },$$

and

(2d) $${\bar{\gamma }}_{\;\;xr}^{\left(m\right)}={\bar{\gamma }}_{\;\;\theta r}^{\left(m\right)}={\bar{\gamma }}_{\;x\theta }^{\left(m\right)}=0,$$

(3a) $${\bar{\sigma }}_{\;xx\;}^{\left(m\right)}=A_{0}\;c_{11}^{\left(m\right)}+\left(c_{12}^{\left(m\right)}/r\right)\;{\bar{W}}_{0}^{\left(m\right)}+c_{\;13}^{\left(m\right)}\;{\bar{W}}_{0}^{\left(m\right)}{,}_{\zeta },$$

(3b) $${\bar{\sigma }}_{\;\theta \theta \;}^{\left(m\right)}=A_{0}\;c_{12}^{\left(m\right)}+\left(c_{22}^{\left(m\right)}/r\right)\;{\bar{W}}_{0}^{\left(m\right)}+c_{\;23}^{\left(m\right)}\;{\bar{W}}_{0}^{\left(m\right)}{,}_{\zeta },$$

(3c) $${\bar{\sigma }}_{\;rr\;}^{\left(m\right)}=A_{0}\;c_{13}^{\left(m\right)}+\left(c_{\;23}^{\left(m\right)}/r\right)\;{\bar{W}}_{0}^{\left(m\right)}+c_{\;33}^{\left(m\right)}\;{\bar{W}}_{0}^{\left(m\right)}{,}_{\zeta },$$

(3d) $${\bar{\sigma }}_{x\;r}^{\left(m\right)}={\bar{\sigma }}_{\theta \;r}^{\left(m\right)}={\bar{\sigma }}_{x\;\theta }^{\left(m\right)}=0,$$

where the commas denote partial differentiation with respect to the suffix variables; the variable $c_{ij}^{\left(m\right)}$ denotes the elastic coefficient.

Selecting the variables ${\bar{W}}_{0}^{\left(m\right)}\;\;\mathrm{and}\;\;{\bar{\sigma }}_{rr}^{\left(m\right)}$ as the state-space variables in the pre-instability analysis and using Equation (3c), we obtain a state-space equation as follows:

(4) $${\bar{W}}_{\;0}^{\left(m\right)}{,}_{\zeta }=-\;{\tilde{c}}_{\;\;13}^{\left(m\right)}A_{0}-\left({\tilde{c}}_{\;\;23}^{\left(m\right)}/r\right)\;{\bar{W}}_{\;\;0}^{\left(m\right)}+\left(1/c_{\;\;33}^{\left(m\right)}\right)\;{\bar{\sigma }}_{\;rr}^{\left(m\right)},$$

where ${\tilde{c}}_{\;k3}^{\left(m\right)}=c_{k\;3}^{\left(m\right)}/c_{\;3\;3}^{\left(m\right)}$ (k=1  and  2), and $m=1,\;2,\cdots ,\;n_{l}$.

With Equations (3a), (3b), and (4), the in-surface normal stresses can be rewritten as follows:

(5) $${\bar{\sigma }}_{\;xx\;}^{\left(m\right)}=\;Q_{\;11}^{\left(m\right)}A_{0}+\left(Q_{12}^{\left(m\right)}/r\right)\;{\bar{W}}_{0}^{\left(m\right)}+{\tilde{c}}_{\;\;13}^{\left(m\right)}\;{\bar{\sigma }}_{\;rr}^{\left(m\right)},$$

(6) $${\bar{\sigma }}_{\;\theta \theta \;}^{\left(m\right)}=Q_{\;12}^{\left(m\right)}A_{0}\;+\left(Q_{\;22}^{\left(m\right)}/r\right)\;{\bar{W}}_{\;0}^{\left(m\right)}+{\tilde{c}}_{\;\;23}^{\left(m\right)}\;{\bar{\sigma }}_{\;rr}^{\left(m\right)},$$

where $Q_{\;ij}^{\left(m\right)}=c_{\;ij}^{\left(m\right)}-\left(c_{\;i\;3}^{\left(m\right)}\;c_{\;j3}^{\left(m\right)}/c_{\;33}^{\left(m\right)}\right)$ (i, j=1  and  2).

According to the displacement model (i.e., Equations (1a)–(1c)) at the pre-instability state, the stress equilibrium equations in the axial and circumferential directions are automatically satisfied, and the stress equilibrium equation in the radial direction can be expressed as follows:

(7) $${\bar{\sigma }}_{r\;r}^{\left(m\right)}{,}_{\zeta }=\left(Q_{\;\;12}^{\left(m\right)}/r\right)A_{0}+\left(Q_{\;\;22}^{\left(m\right)}/r^{2}\right)\;{\bar{W}}_{0}^{\left(m\right)}+\left[\left({\tilde{c}}_{\;\;23}^{\left(m\right)}-1\right)/r\right]\;{\bar{\sigma }}_{\;rr\;}^{\left(m\right)}.$$

Re-organizing Equations (4) and (7), we can obtain a set of state-space equations at the pre-instability state of the cylindrical microshell in the following form:

(8) $$\frac{d\;{\bar{F}}^{\left(m\right)}}{d\;\zeta }\;={\bar{K}}^{\left(m\right)}\;{\bar{F}}^{\left(m\right)}+{\bar{K}}_{p}^{\left(m\right)},$$

where ${\bar{F}}^{\left(m\right)}=\left\{\begin{array}{c} {\bar{W}}_{\;0}^{\left(m\right)}\left(\zeta \right)\\ {\bar{\sigma }}_{\;rr\;}^{\left(m\right)}\left(\zeta \right) \end{array}\right\}$, ${\bar{K}}^{\left(m\right)}=\left[\begin{array}{cc} {\bar{k}}_{\;\;11}^{\left(m\right)} & {\bar{k}}_{\;\;12}^{\left(m\right)}\\ {\bar{k}}_{\;\;21}^{\left(m\right)} & {\bar{k}}_{\;\;22}^{\left(m\right)} \end{array}\right]$, ${\bar{K}}_{p}^{\left(m\right)}=\left\{\begin{array}{c} -\;{\tilde{c}}_{\;\;13}^{\left(m\right)}A_{0}\\ \left(Q_{\;\;12}^{\left(m\right)}/r\right)A_{0}\; \end{array}\right\}$, ${\bar{k}}_{11}^{\left(m\right)}=-{\tilde{c}}_{\;\;23}^{\left(m\right)}/r$, ${\bar{k}}_{12}^{\left(m\right)}=1/c_{\;33}^{\left(m\right)}$, ${\bar{k}}_{\;21}^{\left(m\right)}=Q_{\;\;22}^{\left(m\right)}/r^{2}$, and ${\bar{k}}_{\;22}^{\left(m\right)}=\left({\tilde{c}}_{\;\;23}^{\left(m\right)}-1\right)/r$.

With the stress traction conditions imposed on the outer and inner surfaces of the cylindrical microshell, we can obtain the initial stresses at the pre-instability state by solving Equation (8) using the transfer matrix method combined with the successive approximation method, the detailed description of which was first given by Soldatos and Hadjigeorgiou [40], and also can be found in Wu and Tsai [41] and Wu and Jiang [42].

In addition, the uniform axial strain *A*_0_ can be obtained using the force equilibrium equation at the edges as follows:

Taking a free-body diagram at each edge, we can express the force equilibrium equation in the axial direction as follows:

(9) $$\sum_{m=1}^{n_{l}}\;\int_{\;\theta =0}^{\;\theta =2\;\pi }\;\int_{\;\zeta_{m-1}}^{\;\zeta_{m}}\;{\bar{\sigma }}_{xx}^{\left(m\right)}\left(\zeta \right)\;r\;d\zeta \;d\theta =-P_{\;x}.$$

Substituting Equation (5) into Equation (9), we subsequently obtain the following expression $A_{0}$:

(10) $$A_{0}=\left\{-N_{x}-\left[\sum_{m=1}^{n_{l}}\int_{\zeta_{m-1}}^{\zeta_{m}}\left({\bar{W}}_{\;0}^{\left(m\right)}Q_{\;12}^{\left(m\right)}/r+{\tilde{c}}_{\;\;13}^{\left(m\right)}{\bar{\sigma }}_{\;rr}^{\left(m\right)}\right)\left(1+\zeta /R\right)\;d\zeta \right]\right\}/\left[\sum_{m=1}^{n_{l}}\int_{\zeta_{m-1}}^{\zeta_{m}}Q_{\;11}^{\left(m\right)}\left(1+\zeta /R\right)\;d\zeta \right],$$

where $N_{x}=P_{x}/\left(2\pi R\right)$.

On the one hand, in the case of applying pure axial compression, the value of *A*_0_ can be determined using an iteration scheme between Equations (8) and (10) with an initial guess value of *A*_0_ set at $A_{0}=-1/\left[\sum_{m=1}^{n_{l}}\int_{\zeta_{m-1}}^{\zeta_{m}}Q_{\;11}^{\left(m\right)}\left(1+\zeta /R\right)\;d\zeta \right]$ and the value of *N_x_* at *N_x_* = 1. As a result, the initial normal stresses can be obtained as follows:

(11a) $${\bar{\sigma }}_{\;xx}^{\left(m\right)}\left(\zeta \right)=-a_{\;\;x}^{\left(m\right)}\left(\zeta \right)\;N_{x},$$

(11b) $${\bar{\sigma }}_{\;\theta \theta }^{\left(m\right)}\left(\zeta \right)=-a_{\;\theta }^{\left(m\right)}\left(\zeta \right)\;N_{x},$$

and

(11c) $${\bar{\sigma }}_{\;rr}^{\left(m\right)}\left(\zeta \right)=-a_{\;\;r}^{\left(m\right)}\left(\zeta \right)\;N_{x},$$

where $a_{\;x}^{\left(m\right)}$, $a_{\;\theta }^{\left(m\right)}$, and $a_{\;\;r}^{\left(m\right)}$ denote the influence functions of the initial normal stresses induced in the *m*th layer of the cylindrical microshell at the pre-instability state when the axial compression is applied only.

On the other hand, in the case of applying pure external pressure, the value of *A*_0_ can be determined using an iteration scheme between Equations (8) and (10) with an initial guess value of *A*_0_ set at $A_{0}=0$ and the value of *N_x_* at *N_x_* = 0. As a result, the initial normal stresses can be obtained as follows:

(12a) $${\bar{\sigma }}_{\;xx}^{\left(m\right)}\left(\zeta \right)=-h_{\;\;x}^{\left(m\right)}\left(\zeta \right)\;p_{r},$$

(12b) $${\bar{\sigma }}_{\;\theta \theta }^{\left(m\right)}\left(\zeta \right)=-h_{\;\;\theta }^{\left(m\right)}\left(\zeta \right)\;p_{r},$$

and

(12c) $${\bar{\sigma }}_{\;rr}^{\left(m\right)}\left(\zeta \right)=-h_{\;\;r}^{\left(m\right)}\left(\zeta \right)\;p_{r},$$

where $h_{\;\;x}^{\left(m\right)}$, $h_{\;\;\theta }^{\left(m\right)}$, and $h_{\;\;r}^{\left(m\right)}$ denote the influence functions of the initial normal stresses induced in the *m*th layer of the cylindrical microshell at the pre-instability state when only the external pressure is applied.

## 3. The CCST for Elastic Bodies

As mentioned above, Hadjesfandiari and Dargush [13,14] developed the CCST for analyzing the mechanical behavior of elastic microbodies, where they assumed an asymmetric force-stress tensor ($\sigma_{ij}$) and a skew-symmetric couple-stress tensor ($\mu_{ij}$) induced at a point inside the material of a deformed elastic microbody while considering the couple stress tensor effects. In Hadjesfandiari and Dargush’s CCST, they thus decomposed the force-stress tensor into the symmetric ($\sigma_{\left(ij\right)}$) part and the skew-symmetric ($\sigma_{\left[ij\right]}$) part and distinguished them using parentheses and brackets, respectively, to surround the pair of indices. Then, based on the principle of virtual work, Hadjesfandiari and Dargush expressed the skew-symmetric part of the force-stress tensor in terms of the couple-stress tensor as follows:

(13) $$\sigma_{\left[ji\right]}=-\left(1/2\right)\left(\mu_{i}{,}_{j}-\mu_{j}{,}_{i}\right),$$

where subscripts *i*, *j*, and *k* permute in the natural order and $\mu_{k}=\mu_{ji}=-\mu_{ij}$.

According to Hadjesfandiari and Dargush’s theory, the strain energy density functional of an elastic microbody is a function of the strain tensor ($\varepsilon_{ij}$) and the skew-symmetric part of the curvature tensor ($\kappa_{ij}$), for which $\kappa_{k}=\kappa_{ji}=-\kappa_{ij}$. The strain tensor is a symmetric tensor conjugated with the symmetric part of the force-stress tensor ($\sigma_{\left(ij\right)}$). The constitutive equation between the symmetric part of the stress tensor and the strain tensor is $\sigma_{\left(ij\right)}=c_{ijkl}\;\varepsilon_{kl}$, where $c_{ijkl}$ denotes the elastic coefficient. The skew-symmetric part of the curvature tensor is conjugated with the couple-stress tensor ($\mu_{ij}$), which is skew-symmetric. For an isotropic material, the relationship between the skew-symmetric part of the curvature tensor and the couple-stress tensor is $\mu_{ij}=-8\;Gl^{2}\kappa_{ij}$, where the symbols *G* and *l* denote the shear modulus and the material length-scale parameter. The strain energy stored in the elastic microbody of volume Ω can be expressed as follows:

(14) $$U_{s}=\int_{\Omega }\left[\left(1/2\right)\sigma_{\left(ij\right)}\;\varepsilon_{ij}-\mu_{k}\kappa_{k}\right]\;d\Omega ,$$

where $\varepsilon_{ij}=\left(u_{i}{,}_{j}+u_{j}{,}_{i}\right)/2,$ and $u_{i}$ is the displacement tensor; $\kappa_{k}=\kappa_{ji}=\left(\theta_{j}{,}_{i}-\theta_{i}{,}_{j}\right)/2$, and $\theta_{k}$ denotes the rotation tensor, and $\theta_{k}=\theta_{ji}=\left(u_{j}{,}_{i}-u_{i}{,}_{j}\right)/2$. The detailed expressions for the relationships between the above tensors and the generalized displacement comments are given in the following section.

## 4. The System Equations at the Incremental Perturbation State

### 4.1. The CCST-Based Hermitian C^2^ FLM

#### 4.1.1. Generalized Kinematics Models

As mentioned above, the FG cylindrical microshell is artificially divided into *n_l_* layers with a thickness *h_m_* for the *m*th layer $\left(m=1,\;\;2,\;\ldots ,\;\;n_{l}\right)$, in which the domains of various coordinates are 0≤x≤L, 0≤θ≤2π, $\left(-h_{m}/2\right)\leq z_{m}\leq \left(h_{m}/2\right),$ and $\zeta_{m-1}\leq \zeta \leq \zeta_{m}$. For the Hermitian *C*^2^ FCLM, the elastic displacement components for each layer are expressed as follows:

(15) $$\begin{array}{l} u_{\;\;x}^{\left(m\right)}\left(x,\;\theta ,\;z_{m},\;t\right)=\sum_{i=1}^{n_{d}}\;\left[\psi_{3i-2}^{\left(m\right)}\left(z_{m}\right)u_{\;i}^{\left(m\right)}\left(x,\;\theta ,\;t\right)+\psi_{3i-1}^{\left(m\right)}\left(z_{m}\right)\theta_{\;ui}^{\left(m\right)}\left(x,\;\theta ,\;t\right)+\psi_{3i}^{\left(m\right)}\left(z_{m}\right)\;\kappa_{\;ui}^{\left(m\right)}\left(x,\;\theta ,\;t\right)\right]\\ \;\;\;\;\;\;=\sum_{i=1}^{n_{d}}\;\psi_{\;i}^{\left(m\right)}\;d_{\;ui}^{\left(m\right)}, \end{array}$$

(16) $$\begin{array}{l} u_{\;\;\theta }^{\left(m\right)}\left(x,\;\theta ,\;z_{m},\;t\right)=\sum_{i=1}^{n_{d}}\;\left[\psi_{3i-2}^{\left(m\right)}\left(z_{m}\right)v_{\;i}^{\left(m\right)}\left(x,\;\theta ,\;t\right)+\psi_{3i-1}^{\left(m\right)}\left(z_{m}\right)\theta_{\;vi}^{\left(m\right)}\left(x,\;\theta ,\;t\right)+\psi_{3i}^{\left(m\right)}\left(z_{m}\right)\kappa_{\;vi}^{\left(m\right)}\left(x,\;\theta ,\;t\right)\right]\\ \;\;\;\;\;\;=\sum_{i=1}^{n_{d}}\;\psi_{\;i}^{\left(m\right)}\;d_{\;vi}^{\left(m\right)}, \end{array}$$

(17) $$\begin{array}{l} u_{\;\;r}^{\left(m\right)}\left(x,\;\theta ,\;z_{m},\;t\right)=\sum_{i=1}^{n_{d}}\;\left[\psi_{3i-2}^{\left(m\right)}\left(z_{m}\right)w_{\;i}^{\left(m\right)}\left(x,\;\theta ,\;t\right)+\psi_{3i-1}^{\left(m\right)}\left(z_{m}\right)\theta_{\;wi}^{\left(m\right)}\left(x,\;\theta ,\;t\right)+\psi_{3i}^{\left(m\right)}\left(z_{m}\right)\kappa_{\;wi}^{\left(m\right)}\left(x,\;\theta ,\;t\right)\right]\\ \;\;\;\;\;\;=\sum_{i=1}^{n_{d}}\;\psi_{\;i}^{\left(m\right)}\;d_{\;wi}^{\left(m\right)}, \end{array}$$

where *t* denotes the time variable; *n_d_* denotes the total number of the nodal surfaces for each finite cylindrical layer element; the superscript *m* denotes the *m*th-layer element; $u_{\;i}^{\left(m\right)}$, $v_{\;i}^{\left(m\right)}$, and $w_{\;i}^{\left(m\right)}$ are the elastic displacement components in the *x*, θ, and *r* axes, respectively, on the *i*th nodal surface of the *m*th layer of the microshell; $\theta_{\;\;ui}^{\left(m\right)}$, $\theta_{\;\;vi}^{\left(m\right)}$, and $\theta_{\;\;wi}^{\left(m\right)}$ are the first-order derivatives of $u_{\;i}^{\left(m\right)}$, $v_{\;i}^{\left(m\right)},$ and $w_{\;i}^{\left(m\right)}$ with respect to the thickness coordinate, respectively, such that $\theta_{\;\;fi}^{\left(m\right)}=df_{\;i\;}^{\left(m\right)}/dz_{m},$ where $f_{\;\;i}^{\left(m\right)}=u_{\;\;i}^{\left(m\right)},\;\;v_{\;\;i}^{\left(m\right)},\;\;\mathrm{and}\;\;w_{\;i}^{\left(m\right)}$; $\kappa_{\;\;ui}^{\left(m\right)}$, $\kappa_{\;\;vi}^{\left(m\right)}$, and $\kappa_{\;\;wi}^{\left(m\right)}$ are the second-order derivatives of $u_{\;i}^{\left(m\right)}$, $v_{\;i}^{\left(m\right)}$ and $w_{\;i}^{\left(m\right)}$ with respect to the thickness coordinate, respectively, such that $\kappa_{\;\;fi}^{\left(m\right)}=d^{2}f_{\;i\;}^{\left(m\right)}/dz_{m}^{2}$; $\psi_{\;i}^{\left(m\right)}$ ($i=1,\;\;2,\;\;\ldots ,\;\;3n_{d}$) are the shape (or interpolation) functions which consist of Hermitian *C*^2^ polynomial functions and satisfy the continuity conditions for the first-order and second-order derivatives of each nodal variable; $\psi_{\;i}^{\left(m\right)}=\left\{\begin{array}{cc} \psi_{3i-2}^{\left(m\right)} & \psi_{\;3i-1}^{\left(m\right)}\;\psi_{\;3i}^{\left(m\right)} \end{array}\right\},$ $d_{\;ui}^{\left(m\right)}=\left\{\begin{array}{c} u_{\;i}^{\left(m\right)}\\ \begin{array}{l} \theta_{\;ui}^{\left(m\right)}\\ \kappa_{\;ui}^{\left(m\right)} \end{array} \end{array}\right\},$ $d_{\;vi}^{\left(m\right)}=\left\{\begin{array}{c} v_{\;i}^{\left(m\right)}\\ \begin{array}{l} \theta_{\;vi}^{\left(m\right)}\\ \kappa_{\;vi}^{\left(m\right)} \end{array} \end{array}\right\},$ and $d_{\;wi}^{\left(m\right)}=\left\{\begin{array}{c} w_{\;i}^{\left(m\right)}\\ \begin{array}{l} \theta_{\;wi}^{\left(m\right)}\\ \kappa_{\;wi}^{\left(m\right)} \end{array} \end{array}\right\}.$

For each layer of the FG cylindrical microshell, the linear constitutive equations, which are valid for orthotropic materials, following Hadjesfandiari and Dargush [13,14], are expressed as:

(18) $$\left\{\begin{array}{c} \sigma_{\;\left(xx\right)}^{\left(m\right)}\\ \sigma_{\;\left(\theta \theta \right)}^{\left(m\right)}\\ \sigma_{\left(\;rr\right)}^{\left(m\right)}\\ \sigma_{\left(\theta r\right)}^{\left(m\right)}\\ \sigma_{\left(xr\right)}^{\left(m\right)}\\ \sigma_{\left(x\theta \right)}^{\left(m\right)} \end{array}\right\}=\left[\begin{array}{cccccc} c_{11}^{\left(m\right)} & c_{12}^{\left(m\right)} & c_{13}^{\left(m\right)} & 0 & 0 & 0\\ c_{12}^{\left(m\right)} & c_{22}^{\left(m\right)} & c_{23}^{\left(m\right)} & 0 & 0 & 0\\ c_{13}^{\left(m\right)} & c_{23}^{\left(m\right)} & c_{33}^{\left(m\right)} & 0 & 0 & 0\\ 0 & 0 & 0 & c_{44}^{\left(m\right)} & 0 & 0\\ 0 & 0 & 0 & 0 & c_{55}^{\left(m\right)} & 0\\ 0 & 0 & 0 & 0 & 0 & c_{66}^{\left(m\right)} \end{array}\right]\;\;\left\{\begin{array}{c} \varepsilon_{\;xx}^{\left(m\right)}\\ \varepsilon_{\;\theta \theta }^{\left(m\right)}\\ \varepsilon_{\;rr}^{\left(m\right)}\\ \gamma_{\theta r}^{\left(m\right)}\\ \gamma_{xr}^{\left(m\right)}\\ \gamma_{x\theta }^{\left(m\right)} \end{array}\right\}+\left[\begin{array}{ccc} 0 & 0 & d_{31}^{\left(m\right)}\\ 0 & 0 & d_{32}^{\left(m\right)}\\ 0 & 0 & d_{33}^{\left(m\right)}\\ 0 & d_{24}^{\left(m\right)} & 0\\ d_{15}^{\left(m\right)} & 0 & 0\\ 0 & 0 & 0 \end{array}\right]\;\left\{\begin{array}{c} \kappa_{\;x}^{\left(m\right)}\\ \kappa_{\;\theta }^{\left(m\right)}\\ \kappa_{\;r}^{\left(m\right)} \end{array}\right\},$$

(19) $$\left\{\begin{array}{c} \mu_{\;x}^{\left(m\right)}\\ \mu_{\;\theta }^{\left(m\right)}\\ \mu_{\;r}^{\left(m\right)} \end{array}\right\}=-\left(\frac{1}{2}\right)\left[\begin{array}{cccccc} 0 & 0 & 0 & 0 & d_{15}^{\left(m\right)} & 0\\ 0 & 0 & 0 & d_{24}^{\left(m\right)} & 0 & 0\\ d_{31}^{\left(m\right)} & d_{32}^{\left(m\right)} & d_{33}^{\left(m\right)} & 0 & 0 & 0 \end{array}\right]\;\;\left\{\begin{array}{c} \varepsilon_{\;xx}^{\left(m\right)}\\ \varepsilon_{\;\theta \theta }^{\left(m\right)}\\ \varepsilon_{\;rr}^{\left(m\right)}\\ \gamma_{\theta r}^{\left(m\right)}\\ \gamma_{xr}^{\left(m\right)}\\ \gamma_{x\theta }^{\left(m\right)} \end{array}\right\}-\left(\frac{1}{2}\right)\left[\begin{array}{ccc} b_{11}^{\left(m\right)} & 0 & 0\\ 0 & b_{22}^{\left(m\right)} & 0\\ 0 & 0 & b_{33}^{\left(m\right)} \end{array}\right]\;\left\{\begin{array}{c} \kappa_{\;x}^{\left(m\right)}\\ \kappa_{\;\theta }^{\left(m\right)}\\ \kappa_{\;r}^{\left(m\right)} \end{array}\right\},$$

where $\sigma_{\left(\;xx\right)}^{\left(m\right)},\;\;\sigma_{\left(\theta \theta \right)}^{\left(m\right)},\;\ldots ,\;\;\;\mathrm{and}\;\;\sigma_{\left(x\theta \right)}^{\left(m\right)}$ represent the symmetric parts of the force-stress components; $\mu_{\;x}^{\left(m\right)},\;\;\mu_{\;\;\theta }^{\left(m\right)},\;\;\mathrm{and}\;\;\mu_{\;\;r}^{\left(m\right)}$ are the couple-stress components, for which $\mu_{\;x}^{\left(m\right)}=\mu_{\;r\theta }^{\left(m\right)}=-\mu_{\;\theta r}^{\left(m\right)},$ $\mu_{\;\;\theta }^{\left(m\right)}=\mu_{\;xr}^{\left(m\right)}=-\mu_{\;rx}^{\left(m\right)},$ $\mathrm{and}\;\;\mu_{\;\;r}^{\left(m\right)}=\mu_{\;\theta x}^{\left(m\right)}=-\mu_{\;x\theta }^{\left(m\right)};$ $\varepsilon_{\;xx}^{\left(m\right)},\;\;\varepsilon_{\;\theta \theta }^{\left(m\right)},\;\varepsilon_{\;rr}^{\left(m\right)},$ $\gamma_{\theta r}^{\left(m\right)},\;\;\gamma_{xr}^{\left(m\right)},$ $\mathrm{and}\;\;\gamma_{x\theta }^{\left(m\right)}$ are the strain components; $\kappa_{\;x}^{\left(m\right)},\;\;\kappa_{\;\theta }^{\left(m\right)},\;\;\mathrm{and}$ $\kappa_{\;r}^{\left(m\right)}$ represent the skew-symmetric part of the curvature tensor; $c_{\;ij}^{\left(m\right)},\;\;b_{\;\;kk}^{\left(m\right)},$ and $d_{\;kl}^{\left(m\right)}$ are the elastic coefficients, the material length-scale coefficients, and the coupling force-stress and couple-stress coefficients, respectively. Existing research has never reported experimental data on the coupling force-stress and couple-stress coefficients $\left(d_{\;kl}^{\left(m\right)}\right).$ Thus, we use $d_{\;kl}^{\left(m\right)}$= 0 in this study. The symbols$\;\;b_{\;\;kk}^{\left(m\right)}\;(k=1,\;\;2,\;\;\mathrm{and}\;\;3)$ denote the material length-scale coefficients, defined as $\;b_{\;11}^{\left(m\right)}=16\;G_{r\theta }^{\left(m\right)}\;l_{1}^{2},$ $\;b_{\;22}^{\left(m\right)}=16\;G_{xr}^{\left(m\right)}\;l_{2}^{2},$ and $b_{\;33}^{\left(m\right)}=16\;G_{\;\theta x}^{\left(m\right)}\;l_{3}^{2},$ respectively, where $G_{\;ij}^{\left(m\right)}$ represents the shear modulus related to the *i-j* surface for the *m*th layer and $l_{\;i}^{\left(m\right)}$ is the material length-scale parameter associated with the *k-j* plane for the *m*th layer. When the isotropic material is considered, the coefficients mentioned above are reduced as $b_{\;11}^{\left(m\right)}=b_{\;22}^{\left(m\right)}=b_{\;33}^{\left(m\right)}=16\;Gl^{2}.$

The strain-displacement relationships for each microlayer are given as follows:

(20) $$\varepsilon_{\;xx}^{\left(m\right)}=u_{\;x}^{\left(m\right)}{,}_{x}=\sum_{i=1}^{n_{d}}\;\psi_{\;i}^{\left(m\right)}d_{\;\;ui}^{\left(m\right)}{,}_{x},$$

(21) $$\varepsilon_{\;\theta \theta }^{\left(m\right)}=\left(1/r\right)u_{\;\theta }^{\left(m\right)}{,}_{\theta }+\left(1/r\right)u_{\;r}^{\left(m\right)}=\sum_{i=1}^{n_{d}}\left(1/r\right)\psi_{\;i}^{\left(m\right)}\;d_{\;\;vi\;\;}^{\left(m\right)}{,}_{\theta }+\sum_{i=1}^{n_{d}}\left(1/r\right)\;\psi_{\;i}^{\left(m\right)}\;d_{\;\;wi\;\;}^{\left(m\right)},$$

(22) $$\varepsilon_{\;rr}^{\left(m\right)}=u_{\;\;r}^{\left(m\right)}{,}_{r}=\sum_{i=1}^{n_{d}}\;\left(D\psi_{\;\;i}^{\left(m\right)}\right)\;d_{\;wi}^{\left(m\right)},$$

(23) $$\gamma_{\;xr}^{\left(m\right)}=u_{\;\;x}^{\left(m\right)}{,}_{r}\;+\;u_{\;\;r}^{\left(m\right)}{,}_{x}\;=\sum_{i=1}^{n_{d}}\left(D\psi_{\;i}^{\left(m\right)}\right)\;d_{\;ui}^{\left(m\right)}+\;\sum_{i=1}^{n_{d}}\;\psi_{\;\;i}^{\left(m\right)}\;d_{\;wi}^{\left(m\right)}{,}_{x},$$

(24) $$\gamma_{\;\theta r}^{\left(m\right)}=u_{\;\theta }^{\left(m\right)}{,}_{r}\;-\left(1/r\right)u_{\;\theta }^{\left(m\right)}+\left(1/r\right)\;u_{\;\;r}^{\left(m\right)}{,}_{\theta }=\sum_{i=1}^{n_{d}}\left(D\psi_{\;\;i}^{\left(m\right)}\right)\;d_{\;vi}^{\left(m\right)}-\left(1/r\right)\sum_{i=1}^{n_{d}}\;\psi_{\;\;i}^{\left(m\right)}\;d_{\;\;vi}^{\left(m\right)}+\left(1/r\right)\;\sum_{i=1}^{n_{d}}\;\psi_{\;\;i}^{\left(m\right)}\;d_{\;\;wi}^{\left(m\right)}{,}_{\theta },$$

(25) $$\gamma_{\;x\theta }^{\left(m\right)}=\left(1/r\right)u_{\;x}^{\left(m\right)}{,}_{\theta }\;+\;u_{\;\;\theta }^{\left(m\right)}{,}_{x}=\left(1/r\right)\sum_{i=1}^{n_{d}}\;\psi_{\;i}^{\left(m\right)}\;d_{\;ui}^{\left(m\right)}{,}_{\theta }+\;\sum_{i=1}^{n_{d}}\;\psi_{\;i}^{\left(m\right)}\;d_{\;vi}^{\left(m\right)}{,}_{x},$$

where *m* = 1, 2, …, *n_l_*, and $D\;\psi_{\;i}^{\left(m\right)}=d\;\left(\psi_{\;i}^{\left(m\right)}\right)/dr=d\;\left(\psi_{\;i}^{\left(m\right)}\right)/d\zeta =d\;\left(\psi_{\;i}^{\left(m\right)}\right)/dz_{m}.$

The skew-symmetric parts of the curvatures-to-the displacements relations for each layer are given by

(26) $$\begin{array}{l} \kappa_{\;\;x}^{\left(m\right)}=\left(1/4\right)\left[-\left(1/r^{2}\right)u_{\;\;x}^{\left(m\right)}{,}_{\theta \theta }-\left(1/r\right)u_{\;x}^{\left(m\right)}{,}_{r}-u_{\;x}^{\left(m\right)}{,}_{rr}+\left(1/r\right)u_{\;\;\theta }^{\left(m\right)}{,}_{x\theta }+\left(1/r\right)u_{\;\;r}^{\left(m\right)}{,}_{x}+u_{\;\;r}^{\left(m\right)}{,}_{xr}\right]\\ \;\;\;\;=\left(1/4\right)\left[-\left(1/r^{2}\right)\sum_{i=1}^{n_{d}}\;\psi_{\;i}^{\left(m\right)}\;d_{\;\;ui}^{\left(m\right)}{,}_{\theta \theta }-\left(1/r\right)\sum_{i=1}^{n_{d}}\;\left(D\psi_{\;i}^{\left(m\right)}\right)\;d_{\;\;ui}^{\left(m\right)}-\sum_{i=1}^{n_{d}}\;\left(D^{2}\psi_{\;i}^{\left(m\right)}\right)\;d_{\;\;ui}^{\left(m\right)}\right.\\ \;\;\;\;\;\left.\;\;+\left(1/r\right)\sum_{i=1}^{n_{d}}\;\psi_{\;i}^{\left(m\right)}\;d_{\;\;vi}^{\left(m\right)}{,}_{x\theta }+\left(1/r\right)\sum_{i=1}^{n_{d}}\;\psi_{\;i}^{\left(m\right)}\;d_{\;\;wi}^{\left(m\right)}{,}_{x}+\;\sum_{i=1}^{n_{d}}\;\left(D\psi_{\;i}^{\left(m\right)}\right)\;d_{\;\;wi}^{\left(m\right)}{,}_{x}\right], \end{array}$$

(27) $$\begin{array}{l} \kappa_{\;\;\theta }^{\left(m\right)}=\left(1/4\right)\left[\left(1/r\right)u_{\;\;x}^{\left(m\right)}{,}_{x\theta }-u_{\;\theta }^{\left(m\right)}{,}_{xx}-\left(1/r\right)u_{\;\;\theta }^{\left(m\right)}{,}_{r}-u_{\;\;\theta }^{\left(m\right)}{,}_{rr}+\left(1/r^{2}\right)u_{\;\;\theta }^{\left(m\right)}-\left(1/r^{2}\right)u_{\;\;r}^{\left(m\right)}{,}_{\theta }+\left(1/r\right)u_{\;\;r}^{\left(m\right)}{,}_{\theta r}\right]\\ \;\;\;\;=\left(1/4\right)\left[\left(1/r\right)\sum_{i=1}^{n_{d}}\psi_{\;i}^{\left(m\right)}\;d_{\;\;ui}^{\left(m\right)}{,}_{x\theta }-\right.\sum_{i=1}^{n_{d}}\psi_{\;i}^{\left(m\right)}\;d_{\;\;vi}^{\left(m\right)}{,}_{xx}-\left(1/r\right)\sum_{i=1}^{n_{d}}\left(D\psi_{\;\;i}^{\left(m\right)}\right)\;d_{\;\;vi}^{\left(m\right)}-\sum_{i=1}^{n_{d}}\left(D^{2}\psi_{\;\;i}^{\left(m\right)}\right)d_{\;\;vi}^{\left(m\right)}\;\\ \;\;\;\;\;\;\left.+\left(1/r^{2}\right)\sum_{i=1}^{n_{d}}\psi_{\;i}^{\left(m\right)}\;d_{\;\;vi}^{\left(m\right)}-\left(1/r^{2}\right)\sum_{i=1}^{n_{d}}\psi_{\;i}^{\left(m\right)}\;d_{\;\;wi}^{\left(m\right)}{,}_{\theta }+\left(1/r\right)\sum_{i=1}^{n_{d}}\left(D\psi_{\;\;i}^{\left(m\right)}\right)\;d_{\;\;wi}^{\left(m\right)}{,}_{\theta }\right], \end{array}$$

(28) $$\begin{array}{l} \kappa_{\;\;r}^{\left(m\right)}=\left(1/4\right)\left[u_{\;\;x}^{\left(m\right)}{,}_{xr}+\left(1/r^{2}\right)u_{\;\;\theta }^{\left(m\right)}{,}_{\theta }+\left(1/r\right)u_{\;\;\theta }^{\left(m\right)}{,}_{\theta r}-u_{\;\;r}^{\left(m\right)}{,}_{xx}-\left(1/r^{2}\right)u_{\;\;r}^{\left(m\right)}{,}_{\theta \theta }\right]\\ \;\;\;\;=\left(1/4\right)\left[\sum_{i=1}^{n_{d}}\left(D\psi_{\;i}^{\left(m\right)}\right)d_{\;u\;i}^{\left(m\right)}{,}_{x}+\;\right.\left(1/r^{2}\right)\sum_{i=1}^{n_{d}}\psi_{\;\;i}^{\left(m\right)}\;d_{\;\;vi}^{\left(m\right)}{,}_{\theta }+\left(1/r\right)\sum_{i=1}^{n_{d}}\left(D\psi_{\;i}^{\left(m\right)}\right)d_{\;v\;i}^{\left(m\right)}{,}_{\theta }\;\\ \;\;\;\;\;\;\left.-\sum_{i=1}^{n_{d}}\psi_{\;i}^{\left(m\right)}\;d_{\;\;wi}^{\left(m\right)}{,}_{xx}-\left(1/r^{2}\right)\sum_{i=1}^{n_{d}}\psi_{\;i}^{\left(m\right)}\;d_{\;\;wi}^{\left(m\right)}{,}_{\theta \theta }\right], \end{array}$$

where $D^{2}\psi_{\;i}^{\left(m\right)}=d^{2}\psi_{\;i}^{\left(m\right)}/dr^{2}=d^{2}\psi_{\;i}^{\left(m\right)}/d\zeta^{2}=d^{2}\psi_{\;i}^{\left(m\right)}/dz_{m}^{2}.$

#### 4.1.2. Hamilton’s Principle

The Euler–Lagrange equations of each layer for the Hermitian *C*^2^ FLM can be derived using Hamilton’s principle, and its corresponding energy functional is expressed as follows:

(29) $$I=\sum_{m=1}^{n_{l}}\;\int_{\;t_{1}}^{\;\;t_{2}}\;\left(T^{\left(m\right)}-U_{s}^{\left(m\right)}+W^{\left(m\right)}\right)\;dt$$

where $T^{\left(m\right)},\;\;\;U_{\;s}^{\left(m\right)},\;\;\mathrm{and}\;\;W^{\left(m\right)}$ denote the kinetic energy, the strain energy, and the work done of a typical *m*th layer, respectively, and they are given as follows:

(30) $$T^{\left(m\right)}=\int_{\zeta_{m-1}}^{\zeta_{m}}\;\iint_{\Omega }\;\left(\rho^{\left(m\right)}/2\right)\;\left[{\left(u_{\;x}^{\left(m\right)}{,}_{t}\right)}^{2}+{\left(u_{\;\theta }^{\left(m\right)}{,}_{t}\right)}^{2}+{\left(u_{\;r}^{\left(m\right)}{,}_{t}\right)}^{2}\right]\;r\;dx\;d\theta \;d\zeta ,$$

(31) $$\begin{array}{l} U_{\;s}^{\left(m\right)}=\left(1/2\right)\int_{\zeta_{m-1}}^{\zeta_{m}}\;\iint_{\Omega }\;\left[\sigma_{\left(xx\right)}^{\left(m\right)}\;\varepsilon_{\;xx}^{\left(m\right)}+\sigma_{\left(\theta \theta \right)}^{\left(m\right)}\;\varepsilon_{\;\theta \theta }^{\left(m\right)}+\sigma_{\left(rr\right)}^{\left(m\right)}\;\varepsilon_{rr}^{\left(m\right)}+\sigma_{\left(xr\right)}^{\left(m\right)}\;\gamma_{xr}^{\left(m\right)}+\sigma_{\left(\theta r\right)}^{\left(m\right)}\;\gamma_{\theta r}^{\left(m\right)}+\sigma_{\left(x\theta \right)}^{\left(m\right)}\;\gamma_{\;x\theta }^{\left(m\right)}\right.\\ \;\;\;\;\;\;\;\;\;\;\;\left.\;-2\;\mu_{\;\;x}^{\left(m\right)}\;\kappa_{\;\;x}^{\left(m\right)}-2\;\mu_{\;\;\theta }^{\left(m\right)}\;\kappa_{\;\;\theta }^{\left(m\right)}-2\;\mu_{\;\;r}^{\left(m\right)}\;\kappa_{\;\;r}^{\left(m\right)}\right]\;r\;dx\;d\theta \;d\zeta \;, \end{array}$$

(32) $$W^{\left(m\right)}=\int_{\zeta_{m-1}}^{\zeta_{m}}\;\iint_{\Omega }\;\left\{\left[{\left({\bar{\sigma }}_{\;\left(xx\right)}^{\;\;0}\right)}^{\left(m\right)}\;\varepsilon_{xx}^{nl}+{\left({\bar{\sigma }}_{\;\left(\theta \theta \right)}^{\;\;0}\right)}^{\left(m\right)}\varepsilon_{\theta \theta }^{nl}+{\left({\bar{\sigma }}_{\;\left(rr\right)}^{\;\;0}\right)}^{\left(m\right)}\varepsilon_{rr}^{nl}\right]\right.\;\;r\;dx\;d\theta \;d\zeta ,$$

where Ω denotes the domain of the cylindrical microshell on the x−θ surface; the symbols ${\left({\bar{\sigma }}_{\;\left(xx\right)}^{\;0}\right)}^{\left(m\right)},\;\;{\left({\bar{\sigma }}_{\;\left(\theta \theta \right)}^{\;0}\right)}^{\left(m\right)},$ and ${\left({\bar{\sigma }}_{\;\left(rr\right)}^{\;0}\right)}^{\left(m\right)}$ denote the initial stresses in the *x*, θ, and *r* directions, respectively, at the pre-instability state when the microshell is subjected to combinations of periodic axial compression and periodic external pressure with the intensity $N_{x}$ and *p_r_*, respectively; $\varepsilon_{xx}^{nl},\;\varepsilon_{\theta \theta }^{nl},\;\;\mathrm{and}\;\;\varepsilon_{rr}^{nl}$ are the second-order term of the Green–Lagrange in-surface strains [43]; and

(33a) $${\left({\bar{\sigma }}_{\;xx}^{\;0}\right)}^{\left(m\right)}=-a_{\;x}^{\left(m\right)}\;N_{x}-h_{\;x}^{\left(m\right)}\;p_{r},$$

(33b) $${\left({\bar{\sigma }}_{\;\theta \theta }^{\;0}\right)}^{\left(m\right)}=-a_{\;\theta }^{\left(m\right)}\;N_{x}-h_{\;\theta }^{\left(m\right)}\;p_{r},$$

(33c) $${\left({\bar{\sigma }}_{\;rr}^{\;0}\right)}^{\left(m\right)}=-a_{\;r}^{\left(m\right)}\;N_{x}-h_{\;r}^{\left(m\right)}\;p_{r},$$

(34a) $${\left(\varepsilon_{\;xx}^{nl}\right)}^{\left(m\right)}=\left(1/2\right)\left[{\left(u_{\;\;x}^{\left(m\right)}{,}_{x}\right)}^{2}+{\left(u_{\;\;\theta }^{\left(m\right)}{,}_{x}\right)}^{2}+{\left(u_{\;\;r}^{\left(m\right)}{,}_{x}\right)}^{2}\right],$$

(34b) $${\left(\varepsilon_{\;\theta \theta }^{nl}\right)}^{\left(m\right)}=\left(1/2\right)\left\{{\left[\left(1/r\right)\left(u_{\;\;\theta }^{\left(m\right)}{,}_{\theta }\right)+\left(1/r\right)\left(u_{\;\;r}^{\left(m\right)}\right)\right]}^{2}+{\left[\left(1/r\right)\left(u_{\;\;r}^{\left(m\right)}{,}_{\theta }\right)-\left(1/r\right)\left(u_{\;\;\theta }^{\left(m\right)}\right)\right]}^{2}+{\left[\left(1/r\right)\left(u_{\;\;x}^{\left(m\right)}{,}_{\theta }\right)\right]}^{2}\right\},$$

(34c) $${\left(\varepsilon_{\;rr}^{nl}\right)}^{\left(m\right)}=\left(1/2\right)\left[{\left(u_{\;\;x}^{\left(m\right)}{,}_{r}\right)}^{2}+{\left(u_{\;\;\theta }^{\left(m\right)}{,}_{r}\right)}^{2}+{\left(u_{\;\;r}^{\left(m\right)}{,}_{r}\right)}^{2}\right],$$

where the influence functions $a_{\;\;x}^{\left(m\right)},$ $a_{\;\;\theta }^{\left(m\right)},$ $a_{\;\;r}^{\left(m\right)},$ $h_{\;\;x}^{\;(m)}\;,$ $h_{\;\;\theta }^{\left(m\right)},$ and $h_{\;\;r}^{\left(m\right)}$ are determined using the state-space method and the successive approximation method, of which the solution process is mentioned in Section 2.

In this work, we define a load magnitude ratio between the axial compression and external pressure as follows:

(35) $$\eta =N_{x}/\left(p_{r}\;R\right).$$

As a result, when the external pressure case is considered, the value of η is set at η = 0, and when the axial compression case is considered, the value of η is set at η=∞. When 0<η<∞, the combination of axial compression and external pressure is considered, such that the initial stresses expressed in Equations (33a)–(33c) can be rewritten as follows:

(36a) $${\left({\bar{\sigma }}_{\;xx}^{\;0}\right)}^{\left(m\right)}=-\left[a_{\;\;x}^{\left(m\right)}+h_{\;\;x}^{\left(m\right)}/\left(\eta \;R\right)\;\right]N_{x},$$

(36b) $${\left({\bar{\sigma }}_{\;\theta \theta }^{\;0}\right)}^{\left(m\right)}=-\left[a_{\;\;\theta }^{\left(m\right)}+h_{\;\;e}^{\left(m\right)}/\left(\eta \;R\right)\;\right]\;N_{x},$$

(36c) $${\left({\bar{\sigma }}_{\;rr}^{\;0}\right)}^{\left(m\right)}=-\left[a_{\;\;r}^{\left(m\right)}+h_{\;\;r}^{\left(m\right)}/\left(\eta \;R\right)\;\right]\;N_{x}.$$

As mentioned above, we take the displacement components as the primary variables subject to variation. We first perform the first-order variation of the strain energy, kinetic energy, and work based on the displacement models given in Equations (15)–(17), and then we employ the technique of integration by parts, which results in the following equations:

(37) $$\delta \;U_{\;s}^{\left(m\right)}=\iint_{\Omega }\int_{\zeta_{m-1}}^{\zeta_{m}}\;\left\{{\left(\delta \varepsilon_{\;n}^{\left(m\right)}\right)}^{T}\sigma_{\;n}^{\left(m\right)}+{\left(\delta \varepsilon_{\;s}^{\left(m\right)}\right)}^{T}\sigma_{\;s}^{\left(m\right)}-2{\left(\delta \;\kappa^{\left(m\right)}\right)}^{T}\mu^{\left(m\right)}\right\}\;\;r\;dx\;d\theta \;d\zeta ,$$

(38) $$\delta T^{\left(m\right)}=-\iint_{\Omega }\int_{\zeta_{m-1}}^{\zeta_{m}}\;\rho^{\left(m\right)}\left[\;{\left(\delta \;u^{\left(m\right)}\right)}^{T}{\left({Β}_{\;7}^{\left(m\right)}\right)}^{T}{Β}_{\;7}^{\left(m\right)}u^{\left(m\right)}{,}_{tt}+{\left(\delta \;w^{\left(m\right)}\right)}^{T}{\left({Β}_{\;8}^{\left(m\right)}\right)}^{T}{Β}_{\;8}^{\left(m\right)}w^{\left(m\right)}{,}_{tt}\right]\;r\;dx\;d\theta \;d\zeta ,$$

(39) $$\begin{array}{l} \delta W^{\left(m\right)}=-N_{x}\iint_{\Omega }\int_{\zeta_{m-1}}^{\zeta_{m}}\left[{\left(\delta \;u^{\left(m\right)}\right)}^{T}{\left({Β}_{\;9}^{\left(m\right)}\right)}^{T}g_{\;x}^{\left(m\right)}{Β}_{\;9}^{\left(m\right)}u^{\left(m\right)}+{\left(\delta \;w^{\left(m\right)}\right)}^{T}{\left({Β}_{\;10}^{\left(m\right)}\right)}^{T}g_{\;x}^{\left(m\right)}{Β}_{\;10}^{\left(m\right)}w^{\left(m\right)}\right.\;\\ \;\;\;\;\;\;\;\;\;+{\left(\delta \;u^{\left(m\right)}\right)}^{T}{\left({Β}_{\;11}^{\left(m\right)}\right)}^{T}g_{\;\theta }^{\left(m\right)}\;{Β}_{\;11}^{\left(m\right)}u^{\left(m\right)}+{\left(\delta \;u^{\left(m\right)}\right)}^{T}{\left({Β}_{\;12}^{\left(m\right)}\right)}^{T}g_{\;\theta }^{\left(m\right)}\;{Β}_{\;12}^{\left(m\right)}u^{\left(m\right)}\\ \;\;\;\;\;\;\;\;\;+{\left(\delta \;u^{\left(m\right)}\right)}^{T}{\left({Β}_{\;13}^{\left(m\right)}\right)}^{T}\left(2g_{\;\theta }^{\left(m\right)}\right){Β}_{\;14}^{\left(m\right)}w^{\left(m\right)}+{\left(\delta \;w^{\left(m\right)}\right)}^{T}{\left({Β}_{\;14}^{\left(m\right)}\right)}^{T}\left(2g_{\;\theta }^{\left(m\right)}\right){Β}_{\;13}^{\left(m\right)}u^{\left(m\right)}\\ \;\;\;\;\;\;\;\;\;+{\left(\delta \;w^{\left(m\right)}\right)}^{T}{\left({Β}_{\;14}^{\left(m\right)}\right)}^{T}g_{\;\theta }^{\left(m\right)}\;{Β}_{\;14}^{\left(m\right)}w^{\left(m\right)}+{\left(\delta \;w^{\left(m\right)}\right)}^{T}{\left({Β}_{\;15}^{\left(m\right)}\right)}^{T}g_{\;\theta }^{\left(m\right)}\;{Β}_{\;15}^{\left(m\right)}w^{\left(m\right)}\\ \;\;\;\;\;\;\;\;\;\left.+{\left(\delta \;u^{\left(m\right)}\right)}^{T}{\left({Β}_{\;16}^{\left(m\right)}\right)}^{T}g_{\;r}^{\left(m\right)}\;{Β}_{\;16}^{\left(m\right)}u^{\left(m\right)}+{\left(\delta \;w^{\left(m\right)}\right)}^{T}{\left({Β}_{\;17}^{\left(m\right)}\right)}^{T}g_{\;r}^{\left(m\right)}\;{Β}_{\;17}^{\left(m\right)}w^{\left(m\right)}\right]r\;dx\;d\theta \;d\zeta , \end{array}$$

where the superscript *T* denotes the transposition of the matrix or vector, and the detailed expressions of relevant coefficients, vectors, and matrices in the above Equations (37)–(39) are given in Appendix A.

#### 4.1.3. Layer Element Equations and Structural Equations

The layer element equations for analyzing the 3D dynamic instability behavior of simply-supported FG cylindrical microshells are derived in this section. The microshells of interest are subjected to combinations of periodic axial compression and periodic external pressure, for which

(40) $$N_{x}=\left(\alpha_{s}+\alpha_{d}\;\cos\Omega \;t\right)\;{\left(N_{x}\right)}_{cr},$$

(41) $$p_{r}=\left(\alpha_{s}+\alpha_{d}\;\cos\Omega \;t\right)\;{\left(p_{r}\right)}_{cr},$$

where $\alpha_{s}\;\;\mathrm{and}\;\;\alpha_{d}$ denote the static and dynamic load factors, respectively; Ω represents the excitation frequency. ${\left(N_{x}\right)}_{cr}$ is the critical load for the pure static axial compression case, and ${\left(p_{r}\right)}_{cr}$ is the critical pressure for the pure static external pressure case. In addition, the inequality $\alpha_{s}+\alpha_{d}\leq 1$ is required.

The boundary conditions of each layer at two edges of the microshell are taken to be completely simple supports and are specified as follows:

(42) $$u_{\;\theta }^{\left(m\right)}=u_{\;r}^{\left(m\right)}=\sigma_{\;xx}^{\left(m\right)}=0\;\mathrm{at}\;x=0,\;x=L\;\mathrm{and}\;m=1,\;2,\ldots ,\;n_{l}.$$

The primary variables of each layer are expressed in Equations (15)–(17), and are further expanded as a double Fourier series in the in-surface domain and a harmonic function in the time domain, such that the boundary conditions of the simply-supported edges are exactly satisfied. Thus, the primary variables are expressed as follows:

(43) $$\left[u_{\;i}^{\left(m\right)}\;\theta_{\;ui}^{\left(m\right)}\;\kappa_{\;ui}^{\left(m\right)}\right]=\sum_{\hat{m}=1}^{\infty }\sum_{\hat{n}=0}^{\infty }\;\left[{\left(u_{\;\hat{m}\hat{n}}^{\left(m\right)}\left(t\right)\right)}_{i}\;{\left(\theta_{\;u\hat{m}\hat{n}}^{\left(m\right)}\left(t\right)\right)}_{i}\;{\left(\kappa_{\;u\hat{m}\hat{n}}^{\left(m\right)}\left(t\right)\right)}_{i}\right]\;\;\cos\tilde{m}\;x\;\;\cos\hat{n}\theta ,$$

(44) $$\left[v_{\;i}^{\left(m\right)}\;\theta_{\;vi}^{\left(m\right)}\;\kappa_{\;vi}^{\left(m\right)}\right]=\sum_{\hat{m}=1}^{\infty }\sum_{\hat{n}=0}^{\infty }\;\left[{\left(v_{\;\hat{m}\hat{n}}^{\left(m\right)}\left(t\right)\right)}_{i}\;{\left(\theta_{\;v\hat{m}\hat{n}}^{\left(m\right)}\left(t\right)\right)}_{i}\;{\left(\kappa_{\;v\hat{m}\hat{n}}^{\left(m\right)}\left(t\right)\right)}_{i}\right]\;\;\sin\tilde{m}\;x\;\;\sin\hat{n}\theta ,$$

(45) $$\left[w_{\;i}^{\left(m\right)}\;\theta_{\;wi}^{\left(m\right)}\;\kappa_{\;wi}^{\left(m\right)}\right]=\sum_{\hat{m}=1}^{\infty }\sum_{\hat{n}=0}^{\infty }\;\left[{\left(w_{\;\hat{m}\hat{n}}^{\left(m\right)}\left(t\right)\right)}_{i}\;{\left(\theta_{\;w\hat{m}\hat{n}}^{\left(m\right)}\left(t\right)\right)}_{i}\;{\left(\kappa_{\;w\hat{m}\hat{n}}^{\left(m\right)}\left(t\right)\right)}_{i}\right]\;\;\sin\tilde{m}\;x\;\;\cos\hat{n}\theta ,$$

where $\tilde{m}=\hat{m}\;\pi /L$, and $\hat{m}$ and $\hat{n}$ are the half-wave and full-wave numbers in the *x* and θ directions, respectively.

The layer element equations for the 3D dynamic instability problems of the FG cylindrical microshell can be obtained by introducing Equations (43)–(45) into Equation (29), and then using Hamilton’s principle (i.e., δ Ι=0), which leads to the following equation:

(46) $$\begin{array}{l} \sum_{\hat{m}=1}^{n_{l}}\;\left[\begin{array}{cc} M_{I\;\;I}^{\left(m\right)} & 0\\ 0 & M_{\mathrm{II}\;\;\mathrm{II}}^{\left(m\right)} \end{array}\right]\left\{\begin{array}{c} {\ddot{\tilde{u}}}^{\left(m\right)}\\ {\ddot{\tilde{w}}}^{\left(m\right)} \end{array}\right\}+\sum_{m=1}^{n_{l}}\left\{\left[\begin{array}{cc} K_{I\;\;I}^{\left(m\right)} & K_{I\;\;\mathrm{II}}^{\left(m\right)}\\ K_{\mathrm{II}\;\;I}^{\left(m\right)} & K_{\mathrm{II}\;\;\mathrm{II}}^{\left(m\right)} \end{array}\right]-\left(\alpha_{s}+\alpha_{d}\;\cos\;\Omega \;t\right)\;{\left(N_{x}\right)}_{cr}\left[\begin{array}{cc} A_{I\;\;I}^{\left(m\right)} & A_{I\;\;\mathrm{II}}^{\left(m\right)}\\ A_{\mathrm{II}\;\;I}^{\left(m\right)} & A_{\mathrm{II}\;\;\mathrm{II}}^{\left(m\right)} \end{array}\right]\right.\;\;\\ \;\;\;\;\;\;\left.\;\;\;\;\;\;\;\;-\left(\alpha_{s}+\alpha_{d}\;\cos\;\Omega \;t\right)\;{\left(p_{r}\right)}_{cr}\left[\begin{array}{cc} H_{I\;\;I}^{\left(m\right)} & H_{I\;\;\mathrm{II}}^{\left(m\right)}\\ H_{\mathrm{II}\;\;I}^{\left(m\right)} & H_{\mathrm{II}\;\;\mathrm{II}}^{\left(m\right)} \end{array}\right]\right\}\left\{\begin{array}{c} {\tilde{u}}^{\left(m\right)}\\ {\tilde{w}}^{\left(m\right)} \end{array}\right\}=0, \end{array}$$

where $\left\{\begin{array}{c} \ddot{\tilde{u}}\\ \ddot{\tilde{w}} \end{array}\right\}=\left\{\begin{array}{l} d^{2}\tilde{u}/dt^{2}\\ d^{2}\tilde{w}/dt^{2} \end{array}\right\};$ $K_{k\;\;l}^{\left(m\right)}\left(\psi_{\;i}^{\left(m\right)},\;\;\;\psi_{\;j}^{\left(m\right)}\right)={\left[K_{l\;\;k}^{\left(m\right)}\left(\psi_{\;j}^{\left(m\right)},\;\;\;\psi_{\;i}^{\left(m\right)}\right)\right]}^{T}\;\;\;(k,\;l=I\;\;\mathrm{and}\;\;\mathrm{II});$ $K_{I\;\;I}^{\left(m\right)}=\int_{\zeta_{m-1}}^{\zeta_{m}}\;\left\{{\left({\tilde{B}}_{\;1}^{\left(m\right)}\right)}^{T}\right.r\;Q_{\;cn}^{\left(m\right)}\;{\tilde{B}}_{\;1}^{\left(m\right)}\;+{\left({\tilde{B}}_{\;3}^{\left(m\right)}\right)}^{T}r\;Q_{\;cs}^{\left(m\right)}\;{\tilde{B}}_{\;3}^{\left(m\right)}+\left.{\left({\tilde{B}}_{\;5}^{\left(m\right)}\right)}^{T}r\;Q_{\;b}^{\left(m\right)}\;{\tilde{B}}_{\;5}^{\left(m\right)}\right\}d\zeta ,$ $K_{I\;\;\mathrm{II}}^{\left(m\right)}=\int_{\zeta_{m-1}}^{\zeta_{m}}\;\left\{{\left({\tilde{B}}_{\;1}^{\left(m\right)}\right)}^{T}\right.r\;\;Q_{\;cn}^{\left(m\right)}\;B_{\;2}^{\left(m\right)}\;+{\left({\tilde{B}}_{\;3}^{\left(m\right)}\right)}^{T}r\;Q_{\;cs}^{\left(m\right)}\;{\tilde{B}}_{\;4}^{\left(m\right)}\left.+{\left({\tilde{B}}_{\;5}^{\left(m\right)}\right)}^{T}r\;Q_{\;b}^{\left(m\right)}\;{\tilde{B}}_{\;6}^{\left(m\right)}\right\}d\zeta ,$ $K_{\mathrm{II}\;\;\mathrm{II}}^{\left(m\right)}=\int_{\zeta_{m-1}}^{\zeta_{m}}\;\left\{{\left(B_{\;2}^{\left(m\right)}\right)}^{T}\right.\;r\;Q_{\;cn}^{\left(m\right)}\;B_{\;2}^{\left(m\right)}\;+{\left({\tilde{B}}_{\;4}^{\left(m\right)}\right)}^{T}r\;Q_{\;cs}^{\left(m\right)}\;{\tilde{B}}_{\;4}^{\left(m\right)}+\left.{\left({\tilde{B}}_{\;6}^{\left(m\right)}\right)}^{T}r\;Q_{\;b}^{\left(m\right)}\;{\tilde{B}}_{\;6}^{\left(m\right)}\right\}d\zeta ,$ $A_{k\;\;l}^{\left(m\right)}\left(\psi_{\;i}^{\left(m\right)},\;\;\;\psi_{\;j}^{\left(m\right)}\right)={\left[A_{l\;\;k}^{\left(m\right)}\left(\psi_{\;j}^{\left(m\right)},\;\;\;\psi_{\;i}^{\left(m\right)}\right)\right]}^{T}\;\;\;(k,\;\;l=I\;\;\mathrm{and}\;\;\mathrm{II}),$ $A_{I\;\;I}^{\left(m\right)}=\int_{\zeta_{m-1}}^{\zeta_{m}}\left[{\left({\tilde{B}}_{\;9}^{\left(m\right)}\right)}^{T}{\left(r\;a_{\;x}^{0}\right)}^{\left(m\right)}\;{\tilde{B}}_{\;9}^{\left(m\right)}\;+{\left({\tilde{B}}_{\;11}^{\left(m\right)}\right)}^{T}\;{\left(r\;a_{\;\theta }^{0}\right)}^{\left(m\right)}\;{\tilde{B}}_{\;11}^{\left(m\right)}+{\left(B_{\;12}^{\left(m\right)}\right)}^{T}\;{\left(r\;a_{\;\theta }^{0}\right)}^{\left(m\right)}\;B_{\;12}^{\left(m\right)}+{\left(B_{\;16}^{\left(m\right)}\right)}^{T}\;{\left(ra_{\;r}^{0}\right)}^{\left(m\right)}\;B_{\;16}^{\left(m\right)}\right]\;\;d\zeta ,$ $A_{I\;\;\mathrm{II}}^{\left(m\right)}=\int_{\zeta_{m-1}}^{\zeta_{m}}\left[{\left({\tilde{B}}_{\;13}^{\left(m\right)}\right)}^{T}{\left(2r\;a_{\;\theta }^{0}\right)}^{\left(m\right)}\;B_{\;14}^{\left(m\right)}\;\right]\;\;d\zeta ,$ $A_{\mathrm{II}\;\;\mathrm{II}}^{\left(m\right)}=\int_{\zeta_{m-1}}^{\zeta_{m}}\left[{\left({\tilde{B}}_{\;10}^{\left(m\right)}\right)}^{T}{\left(r\;a_{\;x}^{0}\right)}^{\left(m\right)}\;{\tilde{B}}_{\;10}^{\left(m\right)}\;+{\left(B_{\;14}^{\left(m\right)}\right)}^{T}\;{\left(r\;a_{\;\theta }^{0}\right)}^{\left(m\right)}\;B_{\;14}^{\left(m\right)}+{\left({\tilde{B}}_{\;15}^{\left(m\right)}\right)}^{T}\;{\left(r\;a_{\;\theta }^{0}\right)}^{\left(m\right)}\;{\tilde{B}}_{\;15}^{\left(m\right)}+{\left(B_{\;17}^{\left(m\right)}\right)}^{T}\;{\left(r\;a_{\;r}^{0}\right)}^{\left(m\right)}\;B_{\;17}^{\left(m\right)}\right]\;\;d\zeta ;$ $H_{k\;\;l}^{\left(m\right)}\left(\psi_{\;i}^{\left(m\right)},\;\;\;\psi_{\;j}^{\left(m\right)}\right)={\left[H_{l\;\;k}^{\left(m\right)}\left(\psi_{\;j}^{\left(m\right)},\;\;\;\psi_{\;i}^{\left(m\right)}\right)\right]}^{T}\;\;\;(k,\;l=I\;\;\mathrm{and}\;\;\mathrm{II}),$ $H_{I\;\;I}^{\left(m\right)}=\int_{\zeta_{m-1}}^{\zeta_{m}}\left[{\left({\tilde{B}}_{\;9}^{\left(m\right)}\right)}^{T}{\left(r\;h_{\;x}^{0}\right)}^{\left(m\right)}\;{\tilde{B}}_{\;9}^{\left(m\right)}\;+{\left({\tilde{B}}_{\;11}^{\left(m\right)}\right)}^{T}\;{\left(r\;h_{\;\theta }^{0}\right)}^{\left(m\right)}\;{\tilde{B}}_{\;11}^{\left(m\right)}+{\left(B_{\;12}^{\left(m\right)}\right)}^{T}\;{\left(r\;h_{\;\theta }^{0}\right)}^{\left(m\right)}\;B_{\;12}^{\left(m\right)}+{\left(B_{\;16}^{\left(m\right)}\right)}^{T}\;{\left(r\;h_{\;r}^{0}\right)}^{\left(m\right)}\;B_{\;16}^{\left(m\right)}\right]\;\;d\zeta ,$ $H_{I\;\;\mathrm{II}}^{\left(m\right)}=\int_{\zeta_{m-1}}^{\zeta_{m}}\left[{\left({\tilde{B}}_{\;13}^{\left(m\right)}\right)}^{T}{\left(2r\;h_{\;\theta }^{0}\right)}^{\left(m\right)}\;B_{\;14}^{\left(m\right)}\;\right]\;\;d\zeta ,$ $H_{\mathrm{II}\;\;\mathrm{II}}^{\left(m\right)}=\int_{\zeta_{m-1}}^{\zeta_{m}}\left[{\left({\tilde{B}}_{\;10}^{\left(m\right)}\right)}^{T}{\left(r\;h_{\;x}^{0}\right)}^{\left(m\right)}\;{\tilde{B}}_{\;10}^{\left(m\right)}\;+{\left(B_{\;14}^{\left(m\right)}\right)}^{T}\;{\left(r\;h_{\;\theta }^{0}\right)}^{\left(m\right)}\;B_{\;14}^{\left(m\right)}+{\left({\tilde{B}}_{\;15}^{\left(m\right)}\right)}^{T}\;{\left(r\;h_{\;\theta }^{0}\right)}^{\left(m\right)}\;{\tilde{B}}_{\;15}^{\left(m\right)}+{\left(B_{\;17}^{\left(m\right)}\right)}^{T}\;{\left(r\;h_{\;r}^{0}\right)}^{\left(m\right)}\;B_{\;17}^{\left(m\right)}\right]\;\;d\zeta ;$ $M_{I\;\;I}^{\left(m\right)}=\int_{\zeta_{m-1}}^{\zeta_{m}}\;{\left(B_{\;\;7}^{\left(m\right)}\right)}^{T}\;r\;\rho^{\left(m\right)}\;B_{\;\;7}^{\left(m\right)}\;d\zeta ,$ $M_{\mathrm{II}\;\;\mathrm{II}}^{\left(m\right)}=\int_{\zeta_{m-1}}^{\zeta_{m}}\;{\left(B_{\;8}^{\left(m\right)}\right)}^{T}\;r\;\rho^{\left(m\right)}\;B_{\;8}^{\left(m\right)}\;d\zeta ;$ ${\tilde{u}}^{\left(m\right)}=\left[\begin{array}{c} {\left(d_{u\hat{m}\hat{n}}^{\left(m\right)}\right)}_{i}\\ {\left(d_{v\hat{m}\hat{n}}^{\left(m\right)}\right)}_{i} \end{array}\right]=\left[\begin{array}{c} \begin{array}{l} {\left(u_{\;\hat{m}\hat{n}\;}^{\left(m\right)}\right)}_{i}\\ {\left(\theta_{\;u\hat{m}\hat{n}\;}^{\left(m\right)}\right)}_{i}\\ {\left(\kappa_{\;u\hat{m}\hat{n}\;}^{\left(m\right)}\right)}_{i} \end{array}\\ \begin{array}{l} {\left(v_{\;\hat{m}\hat{n}\;}^{\left(m\right)}\right)}_{i}\\ {\left(\theta_{\;v\hat{m}\hat{n}\;}^{\left(m\right)}\right)}_{i}\\ {\left(\kappa_{\;v\hat{m}\hat{n}\;}^{\left(m\right)}\right)}_{i} \end{array} \end{array}\right],$ ${\tilde{w}}^{\left(m\right)}=\left[{\left(d_{w\hat{m}\hat{n}}^{\left(m\right)}\right)}_{i}\right]=\left[\begin{array}{l} {\left(w_{\;\hat{m}\hat{n}\;\;}^{\left(m\right)}\right)}_{i}\\ {\left(\theta_{\;w\hat{m}\hat{n}\;}^{\left(m\right)}\right)}_{i}\\ {\left(\kappa_{\;w\hat{m}\hat{n}\;}^{\left(m\right)}\right)}_{i} \end{array}\right],$ and the detailed expressions of matrices ${\tilde{B}}_{k}$ are given in Appendix B.

We assemble the element stiffness matrix, the element geometric stiffness matrix, and the element mass matrix for each layer to form the structural stiffness matrix, the structural geometric stiffness matrices, and the structural mass matrix, for which the continuity conditions of each primary variable and its first-order and second-order derivatives with respect to the thickness coordinate are imposed to be satisfied at the nodal surfaces between two adjacent layers, and these are expressed as follows:

(47) $$\begin{array}{l} \left[\begin{array}{cc} M_{11} & 0\\ 0 & M_{22} \end{array}\right]\left\{\begin{array}{c} \ddot{\tilde{u}}\\ \ddot{\tilde{w}} \end{array}\right\}+\left\{\left[\begin{array}{cc} K_{11} & K_{12}\\ K_{21} & K_{22} \end{array}\right]-\left(\alpha_{s}+\alpha_{d}\;\cos\;\Omega \;t\right){\left(N_{x}\right)}_{cr}\left[\begin{array}{cc} A_{11} & A_{12}\\ A_{21} & A_{22} \end{array}\right]\right.\\ \;\;\;\;\;\;\;\;\left.\;\;\;\;\;\;\;-\left(\alpha_{s}+\alpha_{d}\;\cos\;\Omega \;t\right){\left(p_{r}\right)}_{cr}\left[\begin{array}{cc} H_{11} & H_{12}\\ H_{21} & H_{22} \end{array}\right]\right\}\;\;\left\{\begin{array}{c} \tilde{u}\\ \tilde{w} \end{array}\right\}=0. \end{array}$$

As mentioned above, we define the magnitude ratio between the axial compression and the external pressure as η expressed in Equation (35). Afterward, Equation (47) can be rewritten as follows:

(48) $$M\;\ddot{X}+\left\{K-\left(\alpha_{s}+\alpha_{d}\;\cos\;\Omega \;t\right){\left(N_{x}\right)}_{cr}G\;\right\}\;\;X=0,$$

where the matrices **K**, **G**, and **M** denote the stiffness, geometric stiffness, and mass matrices, and $M=\left[\begin{array}{cc} M_{11} & 0\\ 0 & M_{22} \end{array}\right]$, $K=\left[\begin{array}{cc} K_{11} & K_{12}\\ K_{21} & K_{22} \end{array}\right]$, and $G=\left[\begin{array}{cc} G_{11} & G_{12}\\ G_{21} & G_{22} \end{array}\right]$, in which $G_{ij}=A_{ij}+H_{ij}/\left(\eta \;R\right)$, the vector **X** represents the nodal displacements, and $X=\left[\begin{array}{c} \tilde{u}\\ \tilde{w} \end{array}\right]$.

### 4.2. Bolotin’s Method

Equation (48) is a system of Mathieu–Hill equations that govern the dynamic instability behavior of simply-supported FG cylindrical microshells, which are subjected to combinations of periodic axial compression and hydraulic pressure. Bolotin’s method [21] is thus used to find the lowest excitation frequencies for the upper and lower bounds of various principal and secondary dynamic instability regions, for which the nodal displacements are expressed as the infinite terms of periodic functions of time with period T=4π/Ω and T=2π/Ω, respectively, as follows:

For T=4π/Ω,

(49) $$X=\sum_{k=1}^{\infty }\left[a_{2k-1}\;\sin\frac{\left(2k-1\right)\;}{2}\Omega \;t+b_{2k-1}\cos\frac{\left(2k-1\right)\;}{2}\Omega \;t\right].$$

For T=2π/Ω,

(50) $$X=\;b_{0}+\sum_{k=1}^{\infty }\left[a_{2k}\;\sin\;k\;\Omega \;t+b_{2k}\cos k\;\Omega \;t\right].$$

It is well known that the solutions with a period T=4π/Ω are of significant practical importance because the bandwidth of the instability regions obtained using Equation (49) is usually much greater than that obtained using Equation (50). Therefore, the solutions with a period T=4π/Ω are denoted as the principal instability regions, and the solutions with T=2π/Ω are denoted as the secondary instability regions. Furthermore, in the following numerical examples, only the first few terms of Equations (49) and (50) (i.e., *k* = 1, 2, …, *K*) will be adopted due to the rapid convergence of Bolotin’s method.

#### 4.2.1. The Principal Instability Regions

By substituting Equation (49) into Equation (48) and collecting the sine and cosine terms, two sets of linear algebraic equations in $a_{2k-1}$ and $b_{2k-1}$ (*k* = 1, 2, …, *K*) can be formed for each *K*-term solution. The resulting equations are given as follows:

For the one-term solution (*K* = 1),

(51a) $$\sin\;\left(\frac{1}{2}\Omega \;t\right)\;\mathrm{term}:\left[K-\left(\alpha_{s}-\frac{1}{2}\alpha_{d}\right)\;{\left(N_{x}\right)}_{cr}\;G-\frac{\Omega^{2}}{4}\;M\right]\;a_{1}=0;$$

(51b) $$\cos\;\left(\frac{1}{2}\Omega \;t\right)\;\mathrm{term}:\left[K-\left(\alpha_{s}+\frac{1}{2}\alpha_{d}\right)\;{\left(N_{x}\right)}_{cr}\;G-\frac{\Omega^{2}}{4}\;M\right]\;b_{1}=0.$$

For the two-term solution (*K* = 2), $\sin\;\left(\frac{1}{2}\Omega \;t\right)\;\;\mathrm{and}\;\;\sin\;\left(\frac{3}{2}\Omega \;t\right)\mathrm{terms}:$

(52a) $$\left[\begin{array}{cc} K-\left(\alpha_{s}-\frac{1}{2}\alpha_{d}\right)\;{\left(N_{x}\right)}_{cr}\;G-\frac{\Omega^{2}}{4}\;M & \;-\frac{1}{2}\alpha_{d}{\left(N_{x}\right)}_{cr}\;G\\ -\frac{1}{2}\alpha_{d}{\left(N_{x}\right)}_{cr}G & \;K-\alpha_{s}\;{\left(N_{x}\right)}_{cr}\;G-\frac{9\;\Omega^{2}}{4}\;M\; \end{array}\right]\;\left\{\begin{array}{c} a_{1}\\ a_{3} \end{array}\right\}=0;$$

$\cos\;\left(\frac{1}{2}\Omega \;t\right)\;\;\mathrm{and}\;\;\cos\;\left(\frac{3}{2}\Omega \;t\right)\mathrm{terms}:$

(52b) $$\left[\begin{array}{cc} K-\left(\alpha_{s}+\frac{1}{2}\alpha_{d}\right)\;{\left(N_{x}\right)}_{cr}\;G-\frac{\Omega^{2}}{4}\;M & \;-\frac{1}{2}\alpha_{d}{\left(N_{x}\right)}_{cr}\;G\\ -\frac{1}{2}\alpha_{d}{\left(N_{x}\right)}_{cr}\;G & \;K-\alpha_{s}\;{\left(N_{x}\right)}_{cr}\;G-\frac{9\;\Omega^{2}}{4}\;M\; \end{array}\right]\;\left\{\begin{array}{c} b_{1}\\ b_{3} \end{array}\right\}=0.$$

For the *K*-term solution, $\sin\;\left(\frac{1}{2}\Omega \;t\right),\;\;\;\sin\;\left(\frac{3}{2}\Omega \;t\right),\;\;\ldots ,\;\;\mathrm{and}$ $\sin\;\left[\frac{\left(2K-1\right)}{2}\Omega \;t\right]\;\;\mathrm{terms}:$

(53a) $$\left[\begin{array}{cccc} K-\left(\alpha_{s}-\frac{1}{2}\alpha_{d}\right)\;{\left(N_{x}\right)}_{cr}\;G-\frac{\Omega^{2}}{4}\;M & \;\;-\frac{1}{2}\alpha_{d}{\left(N_{x}\right)}_{cr}\;G & \;\ldots & \;0\\ -\frac{1}{2}\alpha_{d}{\left(N_{x}\right)}_{cr}\;G & \;\;K-\alpha_{s}\;{\left(N_{x}\right)}_{cr}\;G-\frac{9\;\Omega^{2}}{4}\;M & \;\ddots & \;0\\ \vdots & \;\;\ddots & \;\ddots & \;-\frac{1}{2}\alpha_{d}{\left(N_{x}\right)}_{cr}\;G\\ 0 & \;\;0 & \;-\frac{1}{2}\alpha_{d}{\left(N_{x}\right)}_{cr}\;G & \;\;K-\alpha_{s}\;{\left(N_{x}\right)}_{cr}\;G-\frac{{\left(2K-1\right)}^{2}\;\Omega^{2}}{4}\;M \end{array}\right]\;\left\{\begin{array}{c} a_{1}\\ \begin{array}{l} \;\;a_{3}\\ \;\;\vdots \\ a_{2K-1} \end{array} \end{array}\right\}=0;$$

$\cos\;\left(\frac{1}{2}\Omega \;t\right),\;\;\;\cos\;\left(\frac{3}{2}\Omega \;t\right),\;\;\ldots ,\;\;\mathrm{and}\;\;\cos\;\left[\frac{\left(2K-1\right)}{2}\Omega \;t\right]\;\;\mathrm{terms}:$

(53b) $$\left[\begin{array}{cccc} K-\left(\alpha_{s}+\frac{1}{2}\alpha_{d}\right)\;{\left(N_{x}\right)}_{cr}\;G-\frac{\Omega^{2}}{4}\;M & \;\;-\frac{1}{2}\alpha_{d}{\left(N_{x}\right)}_{cr}\;G & \;\;\ldots & \;\;0\\ -\frac{1}{2}\alpha_{d}{\left(N_{x}\right)}_{cr}\;G & \;\;K-\alpha_{s}\;{\left(N_{x}\right)}_{cr}\;G-\frac{9\;\Omega^{2}}{4}\;M & \;\;\ddots & \;\;0\\ \vdots & \;\;\ddots & \;\;\ddots & \;\;-\frac{1}{2}\alpha_{d}{\left(N_{x}\right)}_{cr}\;G\\ 0 & \;\;0 & \;\;-\frac{1}{2}\alpha_{d}{\left(N_{x}\right)}_{cr}\;G & \;\;\;K-\alpha_{s}\;{\left(N_{x}\right)}_{cr}\;G-\frac{{\left(2K-1\right)}^{2}\;\Omega^{2}}{4}\;M \end{array}\right]\;\left\{\begin{array}{c} b_{1}\\ \begin{array}{l} \;\;b_{3}\\ \;\;\vdots \\ b_{2K-1} \end{array} \end{array}\right\}=0.$$

For a set of fixed values of $\left(\hat{m},\;\;\hat{n}\right)$, we can obtain the approximate solutions of the lowest excitation frequencies for the upper and lower bounds of the first principal instability region associated with the eigenvectors $a_{1}\;\;\mathrm{and}\;\;b_{1},$ respectively, by setting the determinants of coefficients of Equations (51a) and (51b) at zero. The solutions can then be successively modified using Equations (52a), (52b), (53a), and (53b). Subsequently, we can obtain the approximate solutions of the lowest excitation frequencies for the upper and lower bounds of the second principal instability region associated with the eigenvectors $a_{3}\;\;\mathrm{and}\;\;b_{3},$ respectively, by setting the determinants of coefficients of Equations (52a) and (52b) at zero, and successively modifying these solutions using Equations (53a) and (53b). So on and so forth, the upper and lower bounds of the *k*^th^ principal instability region can also be obtained. Because the bandwidth of the first principal instability region is much larger than the bandwidths of other principal instability regions, only the lowest excitation frequencies for the upper and lower bounds of the first principal instability region of the structure of interest are presented and discussed in the most relevant literature.

#### 4.2.2. The Secondary Instability Regions

By substituting Equation (50) in Equation (48) and collecting the sine and cosine terms, two sets of linear algebraic equations in $a_{2k}$ (*k* = 1, 2, …, *K*) and $b_{2k}$ (*k* = 0, 1, 2, …, *K*) can be formed for each *K*-term solution. The resulting equations are given as follows:

For the one-term solution (*K* = 1),

(54a) $$\sin\;\left(\Omega \;t\right)\mathrm{term}:\left[K-\alpha_{s}{\left(N_{x}\right)}_{cr}G-\Omega^{2}\;M\right]\;a_{2}=0;$$

(54b) $$1\;\;\mathrm{and}\;\;\cos\;\left(\Omega \;t\right)\;\mathrm{term}:\left[\begin{array}{cc} K-\alpha_{s}\;{\left(N_{x}\right)}_{cr}\;G & \;-\alpha_{d}\;{\left(N_{x}\right)}_{cr}\;G/2\\ -\alpha_{d}\;{\left(N_{x}\right)}_{cr}\;G & \;K-\alpha_{s}\;{\left(N_{x}\right)}_{cr}\;G-\Omega^{2}M \end{array}\right]\;\left\{\begin{array}{c} b_{0}\\ b_{2} \end{array}\right\}=0.$$

For the two-term solution (*K* = 2), $\sin\;\left(\Omega \;t\right)\;\;\mathrm{and}\;\;\sin\;\left(2\Omega \;t\right)\mathrm{terms}:$

(55a) $$\left[\begin{array}{cc} K-\alpha_{s}\;{\left(N_{x}\right)}_{cr}\;G-\Omega^{2}\;M & \;-\frac{1}{2}\alpha_{d}{\left(N_{x}\right)}_{cr}\;G\\ -\frac{1}{2}\alpha_{d}{\left(N_{x}\right)}_{cr}\;G & \;K-\alpha_{s}\;{\left(N_{x}\right)}_{cr}\;G-4\Omega^{2}\;M\; \end{array}\right]\;\left\{\begin{array}{c} a_{2}\\ a_{4} \end{array}\right\}=0;$$

$1,\;\;\cos\left(\Omega \;t\right),\;\;\mathrm{and}\;\;\cos\;\left(2\Omega \;t\right)\mathrm{terms}:$

(55b) $$\left[\begin{array}{ccc} K-\alpha_{s}\;{\left(N_{x}\right)}_{cr}\;G & \;\;-\frac{1}{2}\alpha_{d}{\left(N_{x}\right)}_{cr}G & \;\;0\\ -\alpha_{d}{\left(N_{x}\right)}_{cr}G & \;\;K-\alpha_{s}\;{\left(N_{x}\right)}_{cr}\;G-\Omega^{2}\;M & \;\;-\frac{1}{2}\alpha_{d}{\left(N_{x}\right)}_{cr}G\\ 0 & \;\;-\frac{1}{2}\alpha_{d}{\left(N_{x}\right)}_{cr}G & \;\;K-\alpha_{s}\;{\left(N_{x}\right)}_{cr}\;G-4\Omega^{2}\;M \end{array}\right]\;\left\{\begin{array}{c} b_{0}\\ \begin{array}{l} b_{2}\\ b_{4} \end{array} \end{array}\right\}=0.$$

For the *K*-term solution, $\sin\;\left(\Omega \;t\right),\;\;\;\sin\;\left(2\Omega \;t\right),\;\;\ldots ,\;\;\mathrm{and}\;\;\sin\left(K\Omega \;t\right)\;\;\mathrm{terms}:$

(56a) $$\left[\begin{array}{cccc} K-\alpha_{s}\;{\left(N_{x}\right)}_{cr}\;G-\Omega^{2}\;M & -\frac{1}{2}\alpha_{d}{\left(N_{x}\right)}_{cr}\;G & \ldots & 0\\ -\frac{1}{2}\alpha_{d}{\left(N_{x}\right)}_{cr}\;G & K-\alpha_{s}\;{\left(N_{x}\right)}_{cr}\;G-4\Omega^{2}\;M & \ddots & 0\\ \vdots & \ddots & \ddots & -\frac{1}{2}\alpha_{d}{\left(N_{x}\right)}_{cr}\;G\\ 0 & 0 & -\frac{1}{2}\alpha_{d}{\left(N_{x}\right)}_{cr}\;G & \;K-\alpha_{s}\;{\left(N_{x}\right)}_{cr}\;G-K^{2}\;\Omega^{2}M \end{array}\right]\;\left\{\begin{array}{c} a_{2}\\ \begin{array}{l} \;a_{4}\\ \;\;\vdots \\ a_{2K} \end{array} \end{array}\right\}=0;$$

$1,\;\;\cos\;\left(\Omega \;t\right),\;\;\;\cos\;\left(2\Omega \;t\right),\;\;\ldots ,\;\;\mathrm{and}\;\;\cos\left(K\Omega \;t\right)\;\;\mathrm{terms}:$

(56b) $$\left[\begin{array}{cccc} K-\alpha_{s}\;{\left(N_{x}\right)}_{cr}\;G & -\frac{1}{2}\alpha_{d}{\left(N_{x}\right)}_{cr}\;G & \ldots & 0\\ -\alpha_{d}{\left(N_{x}\right)}_{cr}\;G & K-\alpha_{s}\;{\left(N_{x}\right)}_{cr}\;G-\Omega^{2}\;M & \ddots & 0\\ \vdots & \ddots & \ddots & -\frac{1}{2}\alpha_{d}{\left(N_{x}\right)}_{cr}\;G\\ 0 & 0 & -\frac{1}{2}\alpha_{d}{\left(N_{x}\right)}_{cr}\;G & \;K-\alpha_{s}\;{\left(N_{x}\right)}_{cr}\;G-K^{2}\;\Omega^{2}\;M \end{array}\right]\;\left\{\begin{array}{c} b_{0}\\ \begin{array}{l} \;b_{2}\\ \;\;\vdots \\ b_{2K} \end{array} \end{array}\right\}=0.$$

Again, for a set of fixed values of $\left(\hat{m},\;\;\hat{n}\right)$, we can obtain the approximate solutions of the lowest excitation frequency for the upper and lower bounds of the first secondary instability region by setting the determinants of coefficients of Equations (54a) and (54b) at zero, respectively. The solutions can then be successively modified using Equations (55a), (55b), (56a), and (56b). As a result, the approximate solutions of the lowest excitation frequencies for the upper and lower bounds of other secondary instability regions can also be obtained and successively modified by setting the determinants of coefficients of Equations (56a) and (56b) at zero. As mentioned above, the bandwidths of the upper and lower bounds of these secondary instability regions are small compared to the bandwidth of the first principal instability region, leading to the secondary instability regions being ignored in the most relevant literature.

### 4.3. Reduced Cases

On the one hand, Equation (48) represents the system equations for analyzing the 3D free vibration behavior of simply-supported FG cylindrical microshells subjected to combinations of static axial compression and static external pressure before the mechanical instability occurs when we set the value of $\alpha_{d}$ at zero. Furthermore, when the values of both $\alpha_{s}$ and $\alpha_{d}$ are set at zero, Equation (48) can be reduced to the system equations for analyzing the pure 3D free vibration behavior of these microshells. On the other hand, Equation (48) can also be reduced to the system equations for analyzing the static buckling behavior of simply-supported FG cylindrical microshells subjected to combinations of static axial compression and static external pressure when the values of ω, $\alpha_{s}$, and $\alpha_{d}$ are set at zero.

## 5. Numerical Examples

To the best of the authors’ knowledge, no 3D solutions have been reported in the literature for the size-dependent static buckling and dynamic instability behaviors of simply-supported FG cylindrical microshells subjected to combinations of static/periodic axial compression and external pressure. We thus reduce the Hermitian *C*^2^ FLM developed above for analyzing the mechanical behavior of FG cylindrical microshells to the Hermitian *C*^2^ FLM for analyzing that of FG cylindrical macroshells by setting the value of the material length-scale parameter at zero. Afterward, for comparison and validation purposes, we employ the reduced Hermitian *C*^2^ FLM to analyze the static buckling behavior of the macroshells and compare the solutions it produces with the accurate 2D solutions available in the literature. After that, we use the Hermitian *C*^2^ FLM to perform a parametric analysis to realize how some essential factors affect the magnitude of the lowest excitation frequency and its bandwidths of the first principal and first secondary instability regions of the microshells. The material length-scale parameter $\hat{l}$ for the MCST and that of *l* for the CCST are set, following the analysis of Lam et al. [9], at $\hat{l}=2l=17.6\times 10^{-6}\;m.$

### 5.1. Static Buckling

This section presents the results of the comparison and validation studies for the static buckling behavior of simply-supported FG cylindrical macroshells ($\hat{l}/h=0$) and microshells ($\hat{l}/h\neq 0$), which are subjected to combinations of static axial compression and static external pressure.

The accurate solutions of the smallest critical pressure of simply-supported cylindrical isotropic macroshells subjected to static uniform external pressure have been reported in the literature [44,45,46,47,48]. They are thus used to validate the accuracy and convergence rate of the developed Hermitian *C*^2^ FLM. The values of the geometric parameters of the macroshells are *L*/*R* = 1 and 3; *R*/*h* = 300, 500, 1000, and 3000; and *h* = 1 m; additionally, $\hat{l}/h=0.5\;\;\mathrm{and}\;\;1.0,$ for the microshells. The material properties of the macroshells and microshells are *E* = 200 GPa and υ=0.3, where the symbols *E* and υ denote Young’s modulus and Poisson’s ratio, respectively.

Table 1 shows the smallest critical pressure solutions of simply-supported cylindrical isotropic macroshells ($\hat{l}/h=0$) and microshells ($\hat{l}/h=0.5$ and 1.0) obtained using our Hermitian *C*^2^ FLM. Our solutions of the smallest critical pressure of the macroshells are compared with the accurate solutions reported in the literature [44,45,46,47,48]. It can be seen in Table 1 that in the case of macroshells, our solutions of the smallest critical pressure converge rapidly. The convergent solutions of our FLM are obtained when *n_l_* = 2 is used, and are in excellent agreement with the accurate solutions obtained by Vodenitcharova and Ansourian [44] with Flügge’s thin shell theory, by Shen [45] with DCST, and by Sofiyev [46], Khazaeinejad et al. [47], and Mehralian et al. [48] with the FSDT.

In the case of microshells, the results in Table 1 show that the smallest critical pressure increases when the value of the material length-scale parameter becomes greater, which indicates that an increase in the material length-scale parameter causes the microshells to stiffen, which increases their smallest critical pressure. The results also show that the smallest critical pressure of the shells of interest decreases when either the length-to-radius (*L*/*R*) ratio or the radius-to-thickness (*R*/*h*) ratio becomes greater, which indicates an increase in the *L/R* ratio or the *R*/*h* ratio leads to a decrease in the overall stiffness of the shells, which decreases their smallest critical pressure. In addition, the impacts of the length-to-radius (*L*/*R*) ratio and the radius-to-thickness (*R*/*h*) ratio on changes in the wave number pair corresponding to the smallest critical pressure are significant.

Table 2 shows the solutions of our Hermitian *C*^2^ FLM for the smallest critical load of simply-supported single-walled carbon nanotubes (SWCNTs), which are subjected to static axial compression. The values of relevant geometric parameters and material properties are 800 < 2*R*/*h* < 1500; *L/R* = 1; *h* = 1 m and *h* = $17.6\times 10^{-6}\;m$ when $\hat{l}/h$ = 0 and 1.0, respectively; *E* = 1.06 TPa, and υ=0.3. A dimensionless critical load parameter is defined as ${\left({\bar{N}}_{x}\right)}_{cr}=10^{3}{\left(N_{x}\right)}_{cr}/\left(E\;h\right)$. In the case of $\hat{l}/h$ = 0, the relative errors between the solutions obtained using our Hermitian *C*^2^ FCLM and Mehralian and Beni’s solutions with size-dependent LCST [49] and Kim and Kim’s solutions with size-dependent FSDT [50] are less than 1.73% when the values of the 2*R*/*h* ratio are between 800 and 1500, and in the case of $\hat{l}/h$ = 1, the relative errors are less than 1.80%. The results also show that the material length-scale parameter causes the SWCNTs to stiffen, which leads to an increase in the smallest critical load of the SWCNTs.

Table 3 shows the solutions of our Hermitian *C*^2^ FLM for the smallest critical load of simply-supported FG cylindrical macroshells and microshells, which are subjected to combinations of static axial compression and static external pressure. The shells are formed by mixing the metal material (aluminum) and the ceramic material (alumina) according to the volume fractions of the constituent materials, which vary in the thickness direction. The effective material properties of the shells are estimated using the rule of mixtures [3] as follows:

(57a) $$E\left(\zeta \right)=E_{m}\;V_{m}+E_{c}\;V_{c}=E_{m}+\left(E_{c}-E_{m}\right)\;V_{c},$$

(57b) $$\upsilon \left(\zeta \right)=0.3,$$

where the subscripts *m* and *c* denote the metal and ceramic materials, respectively. *V_m_* and *V_c_* are the volume fractions of the metal and ceramic materials, and they are defined as $V_{c}={\left[\left(1/2\right)+\left(\zeta /h\right)\right]}^{\;\kappa_{p}}$ and *V_m_* = 1 − *V_c_*, in which $\kappa_{p}$ denotes the inhomogeneity index. When $\kappa_{p}=0,$ the shells reduce to homogeneous isotropic ceramic shells, and when $\kappa_{p}=\infty ,$ the shells reduce to homogeneous isotropic metal shells.

The values of relevant geometric parameters are *R*/*h* = 10, 100, and 500; *L/R* = 10, and *h* = 1 m for the macroshells. Additionally, $\hat{l}$ = $17.6\times 10^{-6}\;m$ when $\hat{l}/h=0.5\;\;\mathrm{and}\;\;1.0,$ respectively, for microshells. The load magnitude ratio η is 0 (i.e., the pure uniform external pressure case), 1, and 1000 (almost equivalent to the pure axial compression case). Material properties of the aluminum and alumina materials are given as *E_m_* = 70 GPa, *E_c_* = 380 GPa, and $\upsilon_{m}=\upsilon_{c}=0.3.$

It can be seen in Table 3 that the solutions of our Hermitian FLM for the smallest critical load of simply-supported FG cylindrical macroshells subjected to combinations of static axial compression and static external pressure are in excellent agreement with those of Khazaeinejsd et al. [47] with the FSDT. The results also show that the smallest critical load of the shell always occurs when the value of the longitudinal wave number is 1 (i.e., $\hat{m}=1$). In contrast, the value of the circumferential wave number $\hat{n}$ depends on the *L*/*R* and *R*/*h* ratios. The smallest critical load of the shell decreases when the value of the inhomogeneity index $\kappa_{p}$ increases, which indicates that with an increase in the value of $\kappa_{p}$, the overall stiffness of the shells decreases, which in turn decreases the smallest critical load.

### 5.2. Dynamic Instability

Table 4 presents the results of the comparison and validation studies for the first principal instability region of simply-supported homogeneous isotropic cylindrical macroshells subjected to periodic axial compression. The values of relevant geometric parameters are *R*/*h* = 100 and *L/R* = 2, and those of relevant material properties, following Ng et al. [51], are *E* = 211 GPa, υ=0.3, and $\rho =8000\;{\mathrm{kg}/m}^{3}$. The static and dynamic load factors are given as $\alpha_{s}=0.2,$ and $\alpha_{d}=0,\;\;0.1,\;\;0.3,\;\;\mathrm{and}\;\;0.5.$ The wave number pairs considered are $\left(\hat{m},\;\;\hat{n}\right)$ = (1, 2), (1, 3), and (1, 4). The smallest critical load used in this analysis is ${\left({\bar{N}}_{x}\right)}_{cr}=10^{3}{\left(N_{x}\right)}_{cr}/\left(Eh\right)=5.6062,$ which is associated with the wave number pair $\left(\hat{m},\;\;\hat{n}\right)$ = (1, 5). A dimensionless excitation frequency $\bar{\Omega }$ is defined as the same form used in Ng et al. [51] and Sofiyev [28] and given as $\bar{\Omega }=\Omega \;R\;\sqrt{\rho \;\left(1-\upsilon^{2}\right)/E}$.

It can be seen in Table 4 that our Hermitian *C*^2^ solutions of the lowest excitation frequency for the upper and lower bounds of the first principal instability region converge rapidly. The results show that the convergent solutions are yielded when *K* = 2 is used within the ranges considered in this table, i.e., $\hat{m}$ = 1, $\hat{n}$ = 2, 3, and 4; and 0 ≤ α*_d_* ≤ 0.5. The results also show our Hermitian *C*^2^ solutions are in good agreement with those obtained by Ng et al. [51] with the DCST and Sofiyev [28] with the Reddy’s shear deformation theory (RSDT). The relative errors between our Hermitian *C*^2^ solutions and Sofiyev’s RSDT solutions are less than 2.9% for the wave number pairs $\left(\hat{m},\;\;\hat{n}\right)$ = (1, 2) and (1, 3), and the maximum relative error increases to 11.1% for the wave number pair $\left(\hat{m},\;\;\hat{n}\right)$ = (1, 4). This is mainly because the shear deformation effect and the thickness stretching effect become significant when the wave numbers $\hat{m}$ and $\hat{n}$ become greater, which leads to an increase in the difference between the values of the smallest critical load obtained using our Hermitian *C*^2^ FLM and the RSDT.

A parametric analysis examining how some essential factors affect the magnitude of the excitation frequency and its upper and lower bounds for the first principal and first secondary instability regions of simply-supported FG cylindrical microshells subjected to combinations of periodic axial compression and periodic external pressure is presented in the following tables and figures. In particular, the factors considered of interest are the material length-scale parameter, the inhomogeneity index, the load magnitude ratio, the length-to-radius and radius-to-thickness ratios, and the static and dynamic load factors. The microshells of interest are formed by mixing the metal material (aluminum) and the ceramic material (alumina) according to the volume fractions of the constituent materials, which vary in the thickness direction. The effective Young’s modulus and Poisson’s ratio of the microshells are estimated using the rule of mixtures [3] and given in Equations (57a) and (57b), and material properties of the aluminum and alumina materials are given as *E_m_* = 70 GPa, *E_c_* = 380 GPa, and $\upsilon_{m}=\upsilon_{c}=0.3.$

The effective mass density is given as follows:

(58) $$\rho \left(\zeta \right)=\rho_{m}\;V_{m}+\rho_{c}\;V_{c}=\rho_{m}+\left(\rho_{c}-\rho_{m}\right)\;V_{c},$$

where the mass densities of the aluminum and alumina materials are $\rho_{m}=2702\;{\mathrm{kg}/m}^{3}$ and $\rho_{c}=3800\;{\mathrm{kg}/m}^{3}$.

The volume fractions of the alumina and aluminum materials are $V_{c}={\left[\left(1/2\right)+\left(\zeta /h\right)\right]}^{\;\kappa_{p}}$ and *V_m_* = 1 − *V_c_*, respectively. The dimensionless excitation frequency, critical load, and critical pressure are defined in this analysis as $\bar{\Omega }=\Omega \;L^{2}\;\sqrt{\rho {\;}_{m}/E_{m}}/h$, ${\left({\bar{N}}_{x}\right)}_{cr}=10^{3}{\left(N_{x}\right)}_{cr}/\left(E{\;}_{m}h\right)$, and ${\left({\bar{p}}_{r}\right)}_{cr}=10^{3}{\left(p_{r}\right)}_{cr}/E_{m}$, respectively.

**Figure 2:**
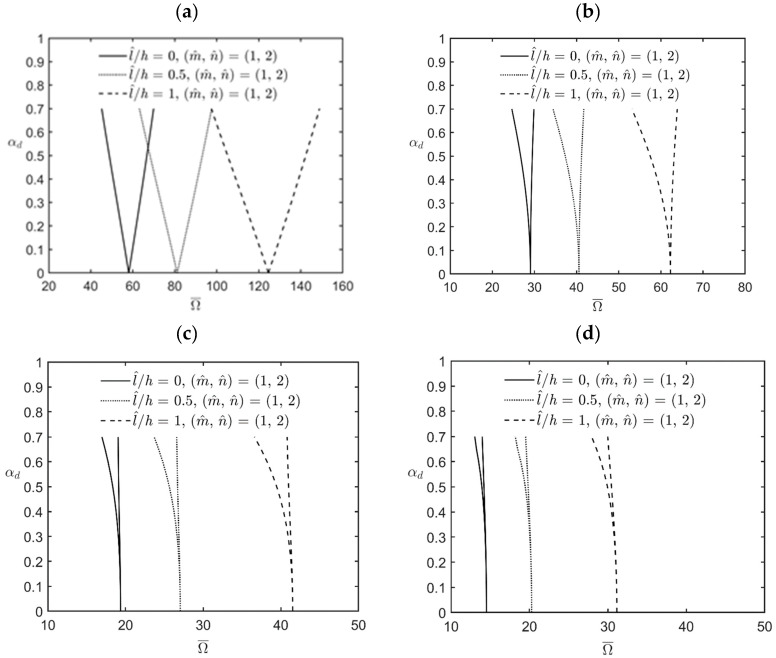
Variations in the lowest excitation frequency for (**a**) the first principal instability region, (**b**) the first secondary instability region, (**c**) the second principal instability region, and (**d**) the second secondary instability region of an FG cylindrical microshell subjected to combinations of periodic axial compression and external load corresponding to changes in the dynamic load factor for different values of the $\hat{l}/h$ ratio.

Figure 2 shows the variations in the lowest excitation frequency for the upper and lower bounds of various instability regions of simply-supported FG cylindrical microshells subjected to combinations of periodic axial compression and periodic external pressure corresponding to changes in the dynamic load factor for different values of the material length-scale parameter. The relevant geometric, material, and load parameters are *R*/*h* = *L*/*R* = 5, $\kappa_{p}$ = 2, $\alpha_{s}=0.2,$ η=2, and $\hat{l}/h=0$, 0.5, and 1. The lowest excitation frequency in the cases of interest occurs when the wave pair number $\left(\hat{m},\;\;\hat{n}\right)$ = (1, 2) is used. Again, it can be seen in Figure 2 that the magnitude of the lowest excitation frequencies and the bandwidths of various instability regions increase when the value of the material length-scale parameter increases, which indicates that an increase in the material length-scale parameter causes the microshells to stiffen, in turn increasing the magnitude of the excitation frequency and the bandwidth of each instability region. For example, for a fixed value of $\alpha_{d}=0.5,$ the bandwidth of the first principal instability region is 66.71 − 48.81 = 17.9 when $\hat{l}/h=0$, while it is 142.38 − 105.16 = 37.22 when $\hat{l}/h=1$. So, the bandwidth of the latter case is about 2.08 times the bandwidth of the former case. On the other hand, the excitation frequency in the middle of the bandwidth is (66.71 + 48.81)/2 = 57.76 when $\hat{l}/h=0$, while it is (142.38 + 105.16)/2 = 123.77 when $\hat{l}/h=1$. So, the excitation frequency of the latter case is about 2.14 times the bandwidth of the former case. The results also show that for a fixed value of the material length-scale parameter, various instability regions can be arranged in the following order by descending bandwidth: the first principal instability region, the first secondary instability region, the second principal instability region, and the second secondary instability region. For example, for a fixed value of $\hat{l}/h=0$, the ratios among the bandwidths of the first principal instability region, the first secondary instability region, the second principal instability region, and the second secondary instability region are 17.91:2.78:0.77:0.26. The bandwidth of the first principal instability region is much greater than those of the other three instability regions. Therefore, most of the published open literature only considered and discussed the excitation frequencies for the upper and lower bounds of the first principal instability region. In the following numerical examples, we present our results of the lowest excitation frequency for the first principal and first secondary instability regions because in some cases, the bandwidth of the first secondary instability region is not too small to be ignored.

**Figure 3:**
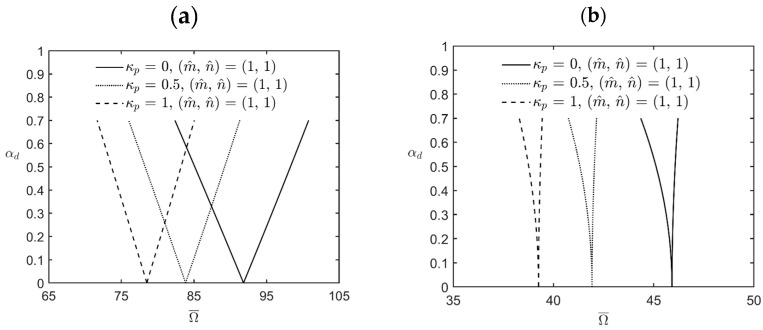
Variations in the lowest excitation frequency for (**a**) the first principal instability region and (**b**) the first secondary instability region of an FG cylindrical microshell subjected to combinations of periodic axial compression and external load corresponding to changes in the dynamic load factor for different values of the inhomogeneity index.

Figure 3 shows the variations in the lowest excitation frequency for the first principal and first secondary instability regions of simply-supported FG cylindrical microshells subjected to combinations of periodic axial compression and periodic external pressure corresponding to changes in the dynamic load factor for different values of the inhomogeneity index $\kappa_{p}$. The relevant geometric, material, and load parameters are *R*/*h* = *L*/*R* = 5, $\alpha_{s}=0.2,$ η = 2, $\hat{l}/h$ = 0.5, and $\kappa_{p}$ = 0, 0.5, and 1. The lowest excitation frequency in the cases of interest occurs when the wave pair number $\left(\hat{m},\;\;\hat{n}\right)$ = (1, 1) is used. It can be seen in Figure 3 that the first principal and secondary instability regions move to the left side, and their bandwidths decrease when the value of the inhomogeneity index $\kappa_{p}$ increases. The reason is that an increase in the value of $\kappa_{p}$ causes the microshell to soften due to the volume fraction of the softer metal material increasing, which leads to smaller bandwidths and lower excitation frequency. For example, for a fixed value of $\alpha_{d}=0.5,$ the bandwidth of the first principal instability region is 98.29 − 85.11 = 13.18 when $\kappa_{p}$ = 0, while it is 83.21 − 73.65 = 9.56 when $\kappa_{p}$ = 1. The bandwidth of the former case is about 1.38 times the bandwidth of the latter case. The magnitude of the excitation frequency in the middle of the first instability region is (98.29 + 85.11)/2 = 91.7 when $\kappa_{p}$ = 0, while it is (83.21 + 73.65)/2 = 78.43 when $\kappa_{p}$ = 1. The lowest excitation frequency for the former case is about 1.17 times the bandwidth of the latter.

**Figure 4:**
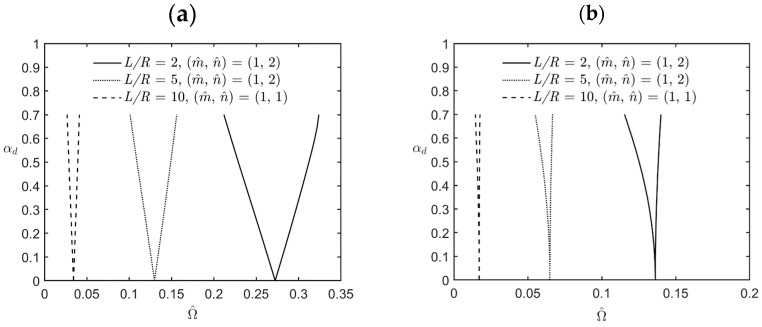
Variations in the lowest excitation frequency for (**a**) the first principal instability region and (**b**) the first secondary instability region of an FG cylindrical microshell subjected to combinations of periodic axial compression and external load corresponding to changes in the dynamic load factor for different values of the *L*/*R* ratio.

Figure 4 shows the variations in the lowest excitation frequency for the first principal and first secondary instability regions of simply-supported FG cylindrical microshells subjected to combinations of periodic axial compression and external pressure corresponding to changes in the dynamic load factor for different values of the *L*/*R* ratio. The relevant geometric, material, and load parameters are *R*/*h* = 5, $\kappa_{p}$ = 2, $\alpha_{s}=0.2,$ η = 2, $\hat{l}/h$ = 0.5, and *L*/*R* = 2, 5, and 10. We redefine a new dimensionless excitation frequency $\bar{\Omega }=\Omega \;h\;\sqrt{\rho {\;}_{m}/E_{m}}$ to make the dimensional and dimensionless excitation frequencies have the same increasing and decreasing trend when the *L*/*R* ratio and the *R*/*h* ratio vary. In the cases of *L*/*R* = 2 and 5, the lowest excitation frequency occurs when the wave pair number $\left(\hat{m},\;\;\hat{n}\right)$ = (1, 2) is used, while it occurs when $\left(\hat{m},\;\;\hat{n}\right)$ = (1, 1) is used in the case of *L*/*R* = 10. It can be seen in Figure 4 that the bandwidth between the upper and lower bounds of the instability regions decreases when the value of the *L*/*R* ratio increases, which indicates that an increase in the value of the *L*/*R* ratio results in a decrease in the overall stiffness of the microshells, in turn decreasing the bandwidth between the upper and lower bounds of the instability regions.

In addition, an increase in the value of the *L*/*R* ratio also decreases the magnitude of the excitation frequency due to a decrease in the overall stiffness of the microshells.

**Figure 5:**
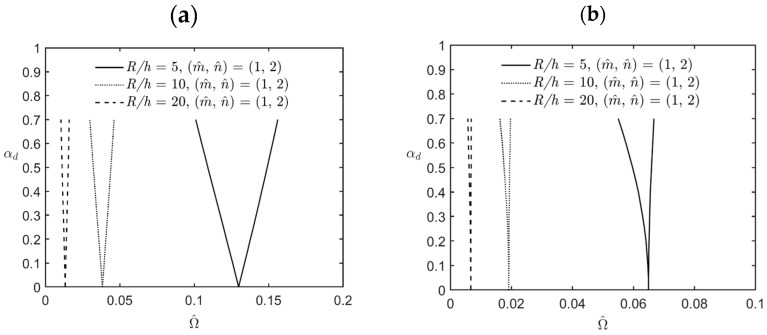
Variations in the lowest excitation frequency for (**a**) the first principal instability region and (**b**) the first secondary instability region of an FG cylindrical microshell subjected to combinations of periodic axial compression and external load corresponding to changes in the dynamic load factor for different values of the *R*/*h* ratio.

Figure 5 shows the variations in the lowest excitation frequency for the first principal and first secondary instability regions of simply-supported FG cylindrical microshells subjected to combinations of periodic axial compression and external pressure corresponding to changes in the dynamic load factor for different values of the *R*/*h* ratio. The relevant geometric, material, and load parameters are *L/R* = 5, $\kappa_{p}$ = 2, $\alpha_{s}=0.2,$ η = 2, $\hat{l}/h$ = 0.5, and *R/h* = 5, 10, and 20. The lowest excitation frequency in these cases occurs when the wave pair number $\left(\hat{m},\;\;\hat{n}\right)$ = (1, 2). It can be seen in Figure 5 that the bandwidth between the upper and lower bounds of the instability regions decreases when the value of the *R*/*h* ratio increases, which indicates that an increase in the value of the *R*/*h* ratio results in a decrease in the overall stiffness of the microshells, decreasing the bandwidth between the upper and lower bounds of the instability regions. In addition, an increase in the value of the *R*/*h* ratio also decreases the magnitude of the excitation frequency due to a decrease in the overall stiffness of the microshells.

**Figure 6:**
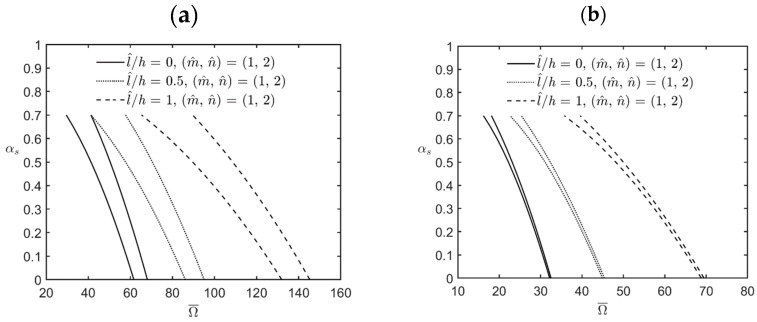
Variations in the lowest excitation frequency for (**a**) the first principal instability region and (**b**) the first secondary instability region of an FG cylindrical microshell subjected to combinations of periodic axial compression and external load corresponding to changes in the static load factor for different values of the $\hat{l}/h$ ratio.

Figure 6 shows the variations in the lowest excitation frequency for the first principal and first secondary instability regions of simply-supported FG cylindrical microshells subjected to combinations of periodic axial compression and periodic external pressure corresponding to changes in the static load factor for different values of the $\hat{l}/h$ ratio. The relevant geometric, material, and load parameters are *R*/*h* = *L*/*R* = 5, $\kappa_{p}$, $\alpha_{d}=0.2$, η = 2, and $\hat{l}/h$ = 0, 0.5, and 1.

The lowest excitation frequency in the cases of interest occurs when the wave pair number $\left(\hat{m},\;\;\hat{n}\right)$ = (1, 2) is used. Again, it can be seen in Figure 2 that both the bandwidths of different instability regions and the excitation frequencies of various instability regions increase when the value of the material length-scale parameter increases, which indicates that an increase in the material length-scale parameter causes the microshells to stiffen, in turn increasing the bandwidth of each instability region and their corresponding excitation frequency.

## 6. Concluding Remarks

In this work, based on the CCST, we developed the Hermitian *C*^2^ FLM to analyze the dynamic instability behavior of simply-supported FG cylindrical microshells subjected to combinations of periodic axial compression and external pressure. Implementing the FLM revealed that the solutions it produced were accurate and converged rapidly, in contrast to the approximate 3D solutions for the static buckling problem of FG cylindrical macroshells available in the literature, for which the value of the material length-scale parameter was set at zero. We also carried out a parametric analysis examining the impacts of some essential factors on the excitation frequency for the upper and lower bounds of FG cylindrical microshells. These factors included the material length-scale parameter, the inhomogeneity index, the load magnitude ratio, the radius-to-thickness and length-to-radius ratios, and the static and dynamic load factors. Conclusions drawn from this parametric study can be summarized as follows:

1. The CCST-based Hermite *C*^2^ FLM for analyzing FG cylindrical microshells can be reduced to those for analyzing FG cylindrical macroshells by setting a zero value to the material length-scale parameter. The static buckling and dynamic instability analyses of the reduced model showed that the CCST-based Hermite *C*^2^ FLM was validated by comparing the solutions it produced with the solutions obtained using the 3D elasticity theory and the 2D advanced shear deformation shell theories reported in the literature.
2. The implementation of Bolotin’s method in the numerical examples showed that convergent solutions were obtained when two terms of the trigonometric functions were used. When arranging by descending order of bandwidth between the upper and lower bounds of excitation frequency, we obtain the following list of instability regions: the first principal instability region, the first secondary instability region, the second principal instability region, and the second secondary instability region.
3. The magnitude of the excitation frequency and its bandwidth between the upper and lower bounds of various instability regions increased when the value of the material length-scale parameter increased, which indicated that an increase in the value of the material length-scale parameter caused the microshell to stiffen, in turn increasing the magnitude of the excitation frequency and its bandwidth for the instability region.
4. The magnitude of the excitation frequency and its bandwidth between the upper and lower bounds of various instability regions decreased when the value of the inhomogeneity index $\kappa_{p}$ increased, which indicates that an increase in the value of $\kappa_{p}$ caused the microshell to soften, in turn decreasing the magnitude of the excitation frequency and its bandwidth for the instability region.
5. The magnitude of the excitation frequency and its bandwidth between the upper and lower bounds of various instability regions decreased when the *L*/*R* ratio or the *R*/*h* ratio increased, which indicates that an increase in either the *L*/*R* ratio or the *R*/*h* ratio results in a decrease in the overall stiffness of the microshells, in turn decreasing the magnitude of the excitation frequency and its bandwidth for the instability region.

To the best of our knowledge, no 3D solutions have yet been proposed for the dynamic instability behavior of FG cylindrical microshells subjected to combinations of axial compression and external pressure. Therefore, the solutions presented in this work can provide a reference for assessing 2D approximate results obtained using CCST-based and MCST-based advanced and refined shell theories.

## Figures and Tables

**Table 1:** Convergence and validation studies for the smallest critical pressure (unit: 10^2^ Pa) of simply-supported cylindrical isotropic macroshells ($\hat{l}/h=0$) and microshells ($\hat{l}/h\neq 0$) under external pressure obtained using the two-node Hermitian *C*^2^ FLMs.

$\hat{l}/h$ **(*l*/*h*)**	Theories	*L*/*R* = 1				*L*/*R* = 3			
		*R*/*h* = 300	*R*/*h* = 500	*R*/*h* = 1000	*R*/*h* = 3000	*R*/*h* = 300	*R*/*h* = 500	*R*/*h* = 1000	*R*/*h* = 3000
0 (0)	Two-node Hermitian *C*^2^ ($n_{l}=1$)	1273.7314	349.4958	60.6715	3.8187	413.8408	115.0668	19.7346	1.2594
	Two-node Hermitian *C*^2^ ($n_{l}=2$)	1273.7314	349.4958	60.6715	3.8188	413.8408	115.0668	19.7345	1.2592
	Vodenitcharova and Ansourian [44]	1269.6	348.43	60.488	3.8100	407.19	NA	NA	1.2510
	Shen [45]	1272.6	348.59	60.536	3.8144	402.60	NA	NA	1.2511
	Sofiyev [46]	1273.5	349.45	60.599	3.8153	412.62	NA	NA	1.2562
	Khazaeinejad et al. [47]	1273.1	349.39	60.595	3.8151	412.57	NA	NA	1.2564
	Mehralian et al. [48]	1273.2	349.40	60.597	3.8152	412.58	NA	NA	1.2561
	$\left(\hat{m},\;\;\hat{n}\right)$	(1, 11)	(1, 13)	(1, 15)	(1, 20)	(1, 7)	(1, 8)	(1, 9)	(1, 12)
0.5	Two-node Hermitian *C*^2^ ($n_{l}=1$)	2219.8700	608.4825	104.7762	6.5912	700.4623	194.2209	33.9787	2.1644
(0.25)	Two-node Hermitian *C*^2^ ($n_{l}=2$)	2219.8700	608.4824	104.7761	6.5912	700.4624	194.2211	33.9785	2.1645
	$\left(\hat{m},\;\;\hat{n}\right)$	(1, 10)	(1, 12)	(1, 14)	(1, 18)	(1, 6)	(1, 7)	(1, 8)	(1, 11)
1.0	Two-node Hermitian *C*^2^ ($n_{l}=1$)	4578.1057	1247.0089	214.3564	13.3529	1467.7834	393.3222	69.1036	4.4041
(0.5)	Two-node Hermitian *C*^2^ ($n_{l}=2$)	4578.1057	1247.0088	214.3564	13.3530	1467.7834	393.3222	69.1036	4.4047
	$\left(\hat{m},\;\;\hat{n}\right)$	(1, 9)	(1, 10)	(1, 12)	(1, 16)	(1, 5)	(1, 6)	(1, 7)	(1, 10)

**Table 2:** Convergence and validation studies with regard to the solutions for the smallest critical load ${\left({\bar{N}}_{x}\right)}_{cr}$ of simply supported, cylindrical macroshells ($\hat{l}$ = 0) and microshells ($\hat{l}\neq 0$) obtained using our two-node Hermitian *C*^2^ FLMs, with different values of the *2R/h* ratio.

2*R*/*h*	$\hat{l}/h$	Numerical Results (Kim and Kim [50])	Analytical Results (Kim and Kim [50])	MCST-Based LCST Results (Mehralian and Beni [49])	CCST-Based FCLMs	
					Hermitian C^2^	Relative Errors
800	0	1.5013	1.5141	1.5131	1.48742	1.73%
	1.0	NA	NA	3.4099	3.34969	1.80%
900	0	1.3586	1.3459	1.3450	1.33319	0.89%
	1.0	NA	NA	3.0464	3.01984	0.88%
1000	0	1.2111	1.2113	1.2105	1.19073	1.66%
	1.0	NA	NA	2.7497	2.71544	1.26%
1100	0	1.1021	1.1012	1.1005	1.08321	1.60%
	1.0	NA	NA	2.4842	2.44885	1.44%
1200	0	1.0170	1.0094	1.0087	1.00143	0.72%
	1.0	NA	NA	2.2783	2.24608	1.43%
1300	0	0.9365	0.9318	0.9311	0.92267	0.91%
	1.0	NA	NA	2.1164	2.08826	1.35%
1400	0	0.8654	0.8652	0.8646	0.85258	1.41%
	1.0	NA	NA	1.9674	1.95456	0.66%
1500	0	0.8075	0.8075	0.8069	0.79602	1.37%
	1.0	NA	NA	1.8309	1.81369	0.95%

Relative errors = 100% × (Mehralian et al.’s solutions–our solutions)/our solutions.

**Table 3:** The smallest critical load solutions of simply supported FG isotropic hollow macrocylinders ($\hat{l}/h=0$) and microcylinders ($\hat{l}/h\neq 0$) subjected to combinations of external pressure and axial compression, and with different length-to-radius and radius-to-thickness ratios ($n_{l}=2$, *L*/*R* = 10).

$\hat{l}/h\;\left(l/h\right)$	Theories	$\kappa_{p}$	*R*/*h* = 10			*R*/*h* = 100			*R*/*h* = 500		
			η=0	η=1	η=1000	η=0	η=1	η=1000	η=0	η=1	η=1000
0 (0)	Two-node Hermitian *C*^2^ FCLM	0	153.078	145.158	1.356	0.383	0.377	0.020	0.0065	0.0065	7.7299 × 10^−4^
	Khazaeinejad et al. [47]		150.244	146.626	NA	0.370	0.366	NA	0.006	0.006	NA
	Two-node Hermitian *C*^2^ FCLM	0.5	100.745	95.414	0.925	0.253	0.250	0.014	0.0044	0.0044	5.2023 × 10^−4^
	Khazaeinejad et al. [47]		97.934	95.575	NA	0.244	0.241	NA	0.004	0.004	NA
	Two-node Hermitian *C*^2^ FCLM	1	78.106	73.924	0.730	0.197	0.194	0.011	0.0035	0.0034	4.0765 × 10^−4^
	Khazaeinejad et al. [47]		75.514	73.696	NA	0.189	0.187	NA	0.003	0.003	NA
	Two-node Hermitian *C*^2^ FCLM	5	50.960	48.204	0.437	0.126	0.124	0.006	0.0021	0.0021	2.5206 × 10^−4^
	Khazaeinejad et al. [47]		49.357	48.169	NA	0.121	0.120	NA	0.002	0.002	NA
	$\left(\hat{m},\;\;\hat{n}\right)$		(1, 2)	(1, 2)	(2, 2)	(1, 3)	(1, 3)	(1, 2)	(1, 4)	(1, 4)	(1, 4)
0.5 (0.25)	Two-node Hermitian *C*^2^ FCLM	0	305.332	289.533	1.483	0.719	0.708	0.023	0.0113	0.0112	0.0011
	Two-node Hermitian *C*^2^ FCLM	0.5	210.961	199.796	1.101	0.498	0.491	0.016	0.0079	0.0078	7.7691 × 10^−4^
	Two-node Hermitian *C*^2^ FCLM	1	167.457	158.489	0.905	0.396	0.390	0.013	0.0063	0.0062	6.2731 × 10^−4^
	$\left(\hat{m},\;\;\hat{n}\right)$		(1, 2)	(1, 2)	(1, 1)	(1, 3)	(1, 3)	(1, 2)	(1, 4)	(1, 4)	(1, 3)
1.0 (0.5)	Two-node Hermitian *C*^2^ FCLM	0	757.117	717.920	1.486	1.728	1.647	0.031	0.0255	0.0253	0.0016
	Two-node Hermitian *C*^2^ FCLM	0.5	537.975	509.495	1.103	1.232	1.190	0.022	0.0182	0.0181	0.0011
	Two-node Hermitian *C*^2^ FCLM	1	432.510	409.343	0.907	0.993	0.965	0.018	0.0147	0.0146	9.1795 × 10^−4^
	$\left(\hat{m},\;\;\hat{n}\right)$		(1, 2)	(1, 2)	(1, 1)	(1, 3)	(1, 2)	(1, 2)	(1, 4)	(1, 4)	(1, 3)

**Table 4:** Comparison study for the upper and lower bounds of the first principal instability region of simply supported homogeneous isotropic cylindrical microshells, which are subjected to periodic axial compression corresponding to changes in the dynamic load factors.

$\left(\hat{m},\;\;\hat{n}\right)$	$\alpha_{d}$	First Principal Instability Region	*K*-Term Approximations					
			*K =* 1		*K =* 2		*K =* 3	
			Cosine Terms	Sine Terms	Cosine Terms	Sine Terms	Cosine Terms	Sine Terms
(1, 2)	0	Hermitian C2 FLM solutions	0.64712	0.64712	0.64712	0.64712	0.64712	0.64712
		Ng et al.’s solutions [51]	0.6485	0.6485	NA	NA	NA	NA
		Sofiyev’s solutions [28]	0.6519	0.6519	NA	NA	NA	NA
	0.1	Hermitian C2 FLM solutions	0.64518	0.64907	0.64518	0.64907	0.64518	0.64907
		Ng et al.’s solutions [51]	0.6481	0.6488	NA	NA	NA	NA
		Sofiyev’s solutions [28]	0.6478	0.6560	NA	NA	NA	NA
	0.3	Hermitian C2 FLM solutions	0.64126	0.65293	0.64127	0.65295	0.64127	0.65295
		Ng et al.’s solutions [51]	0.6475	0.6494	NA	NA	NA	NA
		Sofiyev’s solutions [28]	0.6396	0.6640	NA	NA	NA	NA
	0.5	Hermitian C2 FLM solutions	0.63732	0.65678	0.63736	0.65681	0.63736	0.65681
		Ng et al.’s solutions [51]	0.6468	0.6500	NA	NA	NA	NA
		Sofiyev’s solutions [28]	0.6312	0.6720	NA	NA	NA	NA
(1, 3)	0	Hermitian C2 FLM solutions	0.37843	0.37843	0.37843	0.37843	0.37843	0.37843
		Ng et al.’s solutions [51]	0.3778	0.3778	NA	NA	NA	NA
		Sofiyev’s solutions [28]	0.3719	0.3719	NA	NA	NA	NA
	0.1	Hermitian C2 FLM solutions	0.37509	0.38174	0.37509	0.38175	0.37509	0.38175
		Ng et al.’s solutions [51]	0.3771	0.3784	NA	NA	NA	NA
		Sofiyev’s solutions [28]	0.3696	0.3742	NA	NA	NA	NA
	0.3	Hermitian C2 FLM solutions	0.36831	0.38828	0.36838	0.38834	0.36838	0.38834
		Ng et al.’s solutions [51]	0.3757	0.3796	NA	NA	NA	NA
		Sofiyev’s solutions [28]	0.3649	0.3788	NA	NA	NA	NA
	0.5	Hermitian C2 FLM solutions	0.36141	0.39471	0.36162	0.39487	0.36162	0.39487
		Ng et al.’s solutions [51]	0.3743	0.3807	NA	NA	NA	NA
		Sofiyev’s solutions [28]	0.3601	0.3834	NA	NA	NA	NA
(1, 4)	0	Hermitian C2 FLM solutions	0.24507	0.24507	0.24507	0.24507	0.24507	0.24507
		Ng et al.’s solutions [51]	0.2473	0.2473	NA	NA	NA	NA
		Sofiyev’s solutions [28]	0.2471	0.2471	NA	NA	NA	NA
	0.1	Hermitian C2 FLM solutions	0.23988	0.25015	0.23991	0.25018	0.23991	0.25018
		Ng et al.’s solutions [51]	0.2462	0.2481	NA	NA	NA	NA
		Sofiyev’s solutions [28]	0.2456	0.2487	NA	NA	NA	NA
	0.3	Hermitian C2 FLM solutions	0.22914	0.26002	0.22944	0.26022	0.22944	0.26022
		Ng et al.’s solutions [51]	0.2441	0.2500	NA	NA	NA	NA
		Sofiyev’s solutions [28]	0.2425	0.2517	NA	NA	NA	NA
	0.5	Hermitian C2 FLM solutions	0.21788	0.26953	0.21886	0.27002	0.21886	0.27002
		Ng et al.’s solutions [51]	0.2420	0.2518	NA	NA	NA	NA
		Sofiyev’s solutions [28]	0.2393	0.2547	NA	NA	NA	NA

Hermitian *C*^2^ FLM solutions of the smallest critical load is ${\left({\bar{N}}_{x}\right)}_{cr}=10^{3}{\left(N_{x}\right)}_{cr}/Eh=5.6062$, which are associated with the wave number pair $\left(\hat{m},\;\;\hat{n}\right)$ = (1, 5).

## Data Availability

The processed data required to reproduce these findings are available to download from [https://docs.google.com/document/d/1GL8Aj7_5C4GQgzcuo5O-Msup RtrcqOYQ/edit?usp=drive_link&ouid=103032847656566806520&rtpof=true&sd=true] (Date: 6 January 2024).

## Appendix A. The Detailed Expressions of Relevant Tensors in Equations (37)–(39)

The detailed expressions of relevant coefficients, vectors, and matrices given in Equations (37)–(39) are written as follows:

$$g_{\;\;x}^{\left(m\right)}=a_{\;\;x}^{\left(m\right)}+\left[h_{\;\;x}^{\left(m\right)}/\left(\eta \;R\right)\right],g_{\;\;\theta }^{\left(m\right)}=a_{\;\theta }^{\left(m\right)}+\left[h_{\;\;\theta }^{\left(m\right)}/\left(\eta \;R\right)\right],g_{\;\;r}^{\left(m\right)}=a_{\;\;r}^{\left(m\right)}+\left[h_{\;\;r}^{\left(m\right)}/\left(\eta \;R\right)\right],$$

$$\varepsilon_{\;n}^{\left(m\right)}={\left[\begin{array}{ccc} \varepsilon_{\;xx}^{\left(m\right)} & \varepsilon_{\;\theta \theta }^{\left(m\right)} & \varepsilon_{\;rr}^{\left(m\right)} \end{array}\right]}^{T}=B_{\;1}^{\left(m\right)}\;u^{\left(m\right)}+B_{\;2}^{\left(m\right)}\;w^{\left(m\right)},$$

$$\varepsilon_{\;s}^{\left(m\right)}={\left[\begin{array}{cc} \gamma_{\theta r}^{\left(m\right)} & \;\gamma_{xr}^{\left(m\right)}\;\gamma_{\;x\theta }^{\left(m\right)} \end{array}\right]}^{T}=B_{3}^{\left(m\right)}\;u^{\left(m\right)}+B_{4}^{\left(m\right)}\;w^{\left(m\right)}$$

$$\kappa^{\left(m\right)}={\left[\begin{array}{cc} \kappa_{\;x}^{\left(m\right)} & \;\kappa_{\;\theta }^{\left(m\right)}\;\kappa_{\;\;r}^{\left(m\right)} \end{array}\right]}^{T}=B_{\;5}^{\left(m\right)}\;u^{\left(m\right)}+B_{\;6}^{\left(m\right)}\;w^{\left(m\right)},u^{\left(m\right)}=\left[\begin{array}{c} d_{\;ui\;}^{\left(m\right)}\\ d_{\;vi}^{\left(m\right)} \end{array}\right],\;w^{\left(m\right)}=\left[d_{\;\;wi\;\;}^{\left(m\right)}\right]$$

$$\sigma_{\;n}^{\left(m\right)}={\left[\begin{array}{ccc} \sigma_{\left(x\;x\right)}^{\left(m\right)} & \sigma_{\left(\theta \theta \right)}^{\left(m\right)} & \sigma_{\left(rr\right)}^{\left(m\right)} \end{array}\right]}^{T}=Q_{\;cn}^{\left(m\right)}\;\varepsilon_{\;n}^{\left(m\right)}=Q_{\;cn}^{\left(m\right)}B_{1}^{\left(m\right)}\;u^{\left(m\right)}+Q_{\;cn}^{\left(m\right)}B_{\;2}^{\left(m\right)}\;w^{\left(m\right)},$$

$$\sigma_{\;s}^{\left(m\right)}={\left[\begin{array}{cc} \sigma_{\;\theta r}^{\left(m\right)} & \;\sigma_{\;xr}^{\left(m\right)}\;\sigma_{\;x\theta }^{\left(m\right)} \end{array}\right]}^{T}=Q_{\;cs}^{\left(m\right)}\;\varepsilon_{\;s}^{\left(m\right)}=Q_{\;cs}^{\left(m\right)}\;B_{\;3}^{\left(m\right)}\;u^{\left(m\right)}+Q_{\;cs}^{\left(m\right)}\;B_{\;4}^{\left(m\right)}\;w^{\left(m\right)},$$

$$\mu^{\left(m\right)}={\left[\begin{array}{ccc} \mu_{\;x}^{\left(m\right)} & \mu_{\;\theta }^{\left(m\right)} & \mu_{\;\;r}^{\left(m\right)} \end{array}\right]}^{T}=-\left(1/2\right)Q_{\;b}^{\left(m\right)}\;\kappa^{\left(m\right)}=-\left(1/2\right)\left\{\;Q_{\;b}^{\left(m\right)}B_{\;5}^{\left(m\right)}\;u^{\left(m\right)}\right.\left.+Q_{\;b}^{\left(m\right)}B_{\;6}^{\left(m\right)}\;w^{\left(m\right)}\right\},$$

$$Q_{\;cn}^{\left(m\right)}=\left[\begin{array}{ccc} c_{\;11}^{\left(m\right)} & c_{\;12}^{\left(m\right)} & c_{\;13}^{\left(m\right)}\\ c_{\;12}^{\left(m\right)} & c_{\;22}^{\left(m\right)} & c_{\;23}^{\left(m\right)}\\ c_{\;13}^{\left(m\right)} & c_{\;23}^{\left(m\right)} & c_{\;33}^{\left(m\right)} \end{array}\right],Q_{\;cs}^{\left(m\right)}=\left[\begin{array}{ccc} c_{\;44}^{\left(m\right)} & 0 & 0\\ 0 & c_{\;55}^{\left(m\right)} & 0\\ 0 & 0 & c_{\;66}^{\left(m\right)} \end{array}\right],Q_{\;b}^{\left(m\right)}=\left[\begin{array}{ccc} b_{\;\;11}^{\left(m\right)} & 0 & 0\\ 0 & b_{\;\;22}^{\left(m\right)} & 0\\ 0 & 0 & b_{\;\;33}^{\left(m\right)} \end{array}\right],$$

$$B_{1}^{\left(m\right)}=\left[\begin{array}{cc} \psi_{\;i}^{\left(m\right)}\partial_{x} & 0\\ 0 & \left(\psi_{\;i}^{\left(m\right)}/r\right)\partial_{\theta }\\ 0 & 0 \end{array}\right],B_{\;2}^{\left(m\right)}=\left[\begin{array}{c} 0\\ \psi_{\;i}^{\left(m\right)}/r\\ D\psi_{\;i}^{\left(m\right)} \end{array}\right],$$

$$B_{\;3}^{\left(m\right)}=\left[\begin{array}{cc} 0 & D\psi_{\;i}^{\left(m\right)}-\left(\psi_{\;i}^{\left(m\right)}/r\right)\\ D\psi_{\;i}^{\left(m\right)} & 0\\ \left(\psi_{\;i}^{\left(m\right)}/r\right)\partial_{\theta } & \psi_{\;i}^{\left(m\right)}\partial_{x} \end{array}\right],B_{\;4}^{\left(m\right)}=\left[\begin{array}{c} \left(\psi_{\;i}^{\left(m\right)}/r\right)\;\partial_{\theta }\\ \psi_{\;i}^{\left(m\right)}\;\partial_{x}\\ 0 \end{array}\right],$$

$$B_{\;5}^{\left(m\right)}=\left(1/4\right)\left[\begin{array}{cc} -\left[\left(\psi_{\;i}^{\left(m\right)}/r^{2}\right)\partial_{\theta \theta }+\left(1/r\right)D\psi_{\;i}^{\left(m\right)}+D^{2}\psi_{\;i}^{\left(m\right)}\right] & \left(\psi_{\;i}^{\left(m\right)}/r\right)\partial_{x\theta }\\ \left(\psi_{\;i}^{\left(m\right)}/r\right)\partial_{x\theta } & -\left[\psi_{\;i}^{\left(m\right)}\partial_{xx}+\left(1/r\right)D\psi_{\;i}^{\left(m\right)}+D^{2}\psi_{\;i}^{\left(m\right)}-\left(\psi_{\;i}^{\left(m\right)}/r^{2}\right)\right]\\ \left(D\psi_{\;i}^{\left(m\right)}\right)\partial_{x} & \left(\psi_{\;i}^{\left(m\right)}/r^{2}\right)\partial_{\theta }+\left(D\psi_{\;i}^{\left(m\right)}/r\right)\partial_{\theta } \end{array}\right],$$

$$B_{\;6}^{\left(m\right)}=\left(1/4\right)\left[\begin{array}{c} \left(\psi_{\;i}^{\left(m\right)}/r\right)\;\partial_{x}+\left(D\psi_{\;i}^{\left(m\right)}\right)\;\partial_{x}\\ -\left(\psi_{\;i}^{\left(m\right)}/r^{2}\right)\;\partial_{\theta }+\left(D\psi_{\;i}^{\left(m\right)}/r\right)\;\partial_{\theta }\\ -\left[\psi_{\;i}^{\left(m\right)}\partial_{xx}+\left(\psi_{\;i}^{\left(m\right)}/r^{2}\right)\partial_{\theta \theta }\right] \end{array}\right],B_{\;7}^{\left(m\right)}=\left[\begin{array}{cc} \psi_{\;i}^{\left(m\right)} & 0\\ 0 & \psi_{\;i}^{\left(m\right)} \end{array}\right],B_{\;8}^{\left(m\right)}=\left[\psi_{\;i}^{\left(m\right)}\right],$$

$$B_{\;9}^{\left(m\right)}=\left[\begin{array}{cc} \psi_{\;i}^{\left(m\right)}\partial_{x} & 0\\ 0 & \psi_{\;i}^{\left(m\right)}\partial_{x} \end{array}\right],B_{\;10}^{\left(m\right)}=\left[\psi_{\;i}^{\left(m\right)}\partial_{x}\right],B_{\;11}^{\left(m\right)}=\left[\begin{array}{cc} \left(\psi_{\;i}^{\left(m\right)}/r\right)\partial_{\theta } & 0\\ 0 & \left(\psi_{\;i}^{\left(m\right)}/r\right)\partial_{\theta } \end{array}\right],$$

$$B_{\;12}^{\left(m\right)}=\left[\begin{array}{cc} 0 & \;0\\ 0 & \;\left(\psi_{\;i}^{\left(m\right)}/r\right) \end{array}\right],B_{\;13}^{\left(m\right)}=\left[\begin{array}{cc} 0 & \;0\\ 0 & \;\left(\psi_{\;i}^{\left(m\right)}/r\right)\partial_{\theta } \end{array}\right],B_{\;14}^{\left(m\right)}=\left[\psi_{\;i}^{\left(m\right)}/r\right],$$

$$B_{\;15}^{\left(m\right)}=\left[\left(\psi_{\;i}^{\left(m\right)}/r\right)\partial_{\theta }\right],B_{\;16}^{\left(m\right)}=\left[\begin{array}{cc} D\psi_{i}^{\left(m\right)} & 0\\ 0 & D\psi_{i}^{\left(m\right)} \end{array}\right],B_{\;17}^{\left(m\right)}=\left[D\psi_{\;i}^{\left(m\right)}\right];$$

and $i=1,\;2,\cdots ,\;n_{d}$, and $m=1,\;2,\cdots ,\;n_{l}$.

## Appendix B. The Detailed Expressions of Matrices ${\tilde{B}}_{k}$

The detailed expressions of matrices ${\tilde{B}}_{k}$ are given as follows:

$${\tilde{B}}_{1}^{\left(m\right)}=\left[\begin{array}{cc} -\tilde{m}\;\psi_{\;i}^{\left(m\right)} & 0\\ 0 & \hat{n}\left(\psi_{\;i}^{\left(m\right)}/r\right)\\ 0 & 0 \end{array}\right],{\tilde{B}}_{\;3}^{\left(m\right)}=\left[\begin{array}{cc} 0 & D\psi_{\;i}^{\left(m\right)}-\left(\psi_{\;i}^{\left(m\right)}/r\right)\\ D\psi_{\;i}^{\left(m\right)} & 0\\ -\hat{n}\left(\psi_{\;i}^{\left(m\right)}/r\right) & \tilde{m}\;\psi_{\;i}^{\left(m\right)} \end{array}\right],{\tilde{B}}_{\;4}^{\left(m\right)}=\left[\begin{array}{c} -\hat{n}\;\left(\psi_{\;i}^{\left(m\right)}/r\right)\;\\ \tilde{m}\;\psi_{\;i}^{\left(m\right)}\;\\ 0 \end{array}\right],$$

$${\tilde{B}}_{\;5}^{\left(m\right)}=\left(1/4\right)\left[\begin{array}{cc} \left[{\hat{n}}^{2}\left(\psi_{\;i}^{\left(m\right)}/r^{2}\right)-\left(1/r\right)D\psi_{\;i}^{\left(m\right)}-D^{2}\psi_{\;i}^{\left(m\right)}\right] & \;\tilde{m}\;\hat{n}\left(\psi_{\;i}^{\left(m\right)}/r\right)\\ \tilde{m}\;\hat{n}\left(\psi_{\;i}^{\left(m\right)}/r\right) & \;\left[{\tilde{m}}^{2}\psi_{\;i}^{\left(m\right)}-\left(1/r\right)D\psi_{\;i}^{\left(m\right)}-D^{2}\psi_{\;i}^{\left(m\right)}+\left(\psi_{\;i}^{\left(m\right)}/r^{2}\right)\right]\\ -\tilde{m}\left(D\psi_{\;i}^{\left(m\right)}\right) & \;\hat{n}\left(\psi_{\;i}^{\left(m\right)}/r^{2}\right)+\hat{n}\left(D\psi_{\;i}^{\left(m\right)}/r\right) \end{array}\right],$$

$${\tilde{B}}_{\;6}^{\left(m\right)}=\left(1/4\right)\left[\begin{array}{c} \tilde{m}\left(\psi_{\;i}^{\left(m\right)}/r\right)\;+\tilde{m}\left(D\psi_{\;i}^{\left(m\right)}\right)\;\\ \hat{n}\left(\psi_{\;i}^{\left(m\right)}/r^{2}\right)\;-\hat{n}\left(D\psi_{\;i}^{\left(m\right)}/r\right)\;\\ {\tilde{m}}^{2}\psi_{\;i}^{\left(m\right)}+{\hat{n}}^{2}\left(\psi_{\;i}^{\left(m\right)}/r^{2}\right) \end{array}\right],{\tilde{B}}_{\;9}^{\left(m\right)}=\left[\begin{array}{cc} -\tilde{m}\;\psi_{\;i}^{\left(m\right)} & 0\\ 0 & \tilde{m}\;\psi_{\;i}^{\left(m\right)} \end{array}\right],{\tilde{B}}_{\;10}^{\left(m\right)}=\left[\tilde{m}\;\psi_{\;i}^{\left(m\right)}\right],$$

$${\tilde{B}}_{\;11}^{\left(m\right)}=\left[\begin{array}{cc} -\hat{n}\;\left(\psi_{\;i}^{\left(m\right)}/r\right) & 0\\ 0 & \hat{n}\left(\psi_{\;i}^{\left(m\right)}/r\right) \end{array}\right],{\tilde{B}}_{\;13}^{\left(m\right)}=\left[\begin{array}{cc} 0 & \;0\\ 0 & \;\hat{n}\left(\psi_{\;i}^{\left(m\right)}/r\right) \end{array}\right],\;\mathrm{and}\;{\tilde{B}}_{\;15}^{\left(m\right)}=\left[-\hat{n}\left(\psi_{\;i}^{\left(m\right)}/r\right)\right].$$

## References

[1] Koizumi M. (1997). FGM activities in Japan. Compos. Part B.

[2] Miyamoto Y., Kaysser W.A., Rabin B.H., Kawasaki A., Ford R.G. (1999). Functionally Graded Materials: Design, Proceeding and Applications.

[3] Shen H.S. (2009). Functionally Graded Materials, Nonlinear Analysis of Plates and Shells.

[4] Wu C.P., Li K.W. (2021). Multi-objective optimization of functionally graded beams using a genetic algorithm with non-dominated sorting. J. Compos. Sci..

[5] Ding S., Wu C.P. (2018). Optimization of material composition to minimize the thermal stresses induced in FGM plates with temperature-dependent material properties. Int. J. Mech. Mater. Des..

[6] Bharti I., Gupta N., Gupta K.M. (2013). Novel applications of functionally graded nano, opto-electric and thermo-electric materials. Int. J. Mater. Mech. Manufact..

[7] Faudzi A.A.M., Sabzehmeidani Y., Suzumori K. (2020). Application of micro-electro-mechanical systems (MEMS) as sensors: A review. J. Robot. Mechatr..

[8] Hierold C., Jungen A., Srampfer C., Helbling T. (2007). Nano electromechanical sensors based on carbon nanotubes. Sens. Actuators A Phys..

[9] Lam D.C.C., Yang F., Chong A.C.M., Wang J., Tong P. (2003). Experiments and theory in strain gradient elasticity. J. Mech. Phys. Solids.

[10] Wisnom M.R. (1999). Size effects in the testing of fibre-composite materials. Compos. Sci. Technol..

[11] McFarland A.W., Colton J.S. (2005). Role of material microstructure in plate stiffness with relevance to microcantilever sensors. J. Micromech. Microeng..

[12] Yang F., Chong A.C.M., Lam D.C.C., Tong P. (2002). Couple stress-based strain gradient theory for elasticity. Int. J. Solids Struct..

[13] Hadjesfandiari A.R., Dargush G.F. (2011). Couple stress theory for solids. Int. J. Solids Struct..

[14] Hadjesfandiari A.R., Dargush G.F. (2013). Fundamental solutions for isotropic size-dependent couple stress elasticity. Int. J. Solids Struct..

[15] Koiter W.T. (1964). Couple stresses in the theory of elasticity, I and II. Proc. Roy. Netherlands Acad. Arts Sci..

[16] Mindlin R.D., Tiersten H.F. (1962). Effects of couple-stresses in linear elasticity. Arch. Ration. Mech. Anal..

[17] Toupin R.A. (1962). Elastic materials with couple-stresses. Arch. Ration. Mech. Anal..

[18] Argento A., Scott R.A. (1993). Dynamic instability of layered anisotropic circular cylindrical shells, Part I: Theoretical development. J. Sound Vibr..

[19] Argento A., Scott R.A. (1993). Dynamic instability of layered anisotropic circular cylindrical shells, Part II: Numerical results. J. Sound Vibr..

[20] Xie W.C. (2006). Dynamic Stability of Structures.

[21] Bolotin V.V. (1964). The Dynamic Stability of Elastic Systems.

[22] Ganapathi M., Balamurugan V. (1998). Dynamic instability analysis of a laminated composite circular cylindrical shell. Comput. Struct..

[23] Ganapathi M., Patel B.P. (1999). Parametric dynamic instability analysis of laminated composite conical shells. J. Reinf. Plast. Compos..

[24] Sofiyev A.H., Pancar E.B. (2017). The effect of heterogeneity on the parametric instability of axially excited orthotropic conical shells. Thin-Walled Struct..

[25] Bert C.W., Birman V. (1988). Parametric instability of thick, orthotropic, circular cylindrical shells. Acta Mech..

[26] Ng T.Y., Hua L., Lam K.Y., Loy C.T. (1999). Parametric instability of conical shells by the generalized differential quadrature method. Int. J. Numer. Methods Eng..

[27] Wu C.P., Chiu S.J. (2002). Thermally induced dynamic instability of laminated composite conical shells. Int. J. Solids Struct..

[28] Sofiyev A.H. (2015). Influences of shear stresses on the dynamic instability of exponentially graded sandwich cylindrical shells. Compos. Part B.

[29] Sofiyev A.H. (2016). Parametric vibration of FGM conical shells under periodic lateral pressure within the shear deformation theory. Compos. Part B.

[30] Ganapathi M., Patel B.P., Sambandam C.T., Touratier M. (1999). Dynamic instability analysis of circular conical shells. Compos. Struct..

[31] Pradyumna S., Bandyopadhyay J.N. (2010). Dynamic instability of functionally graded shells using higher-order theory. J. Eng. Mech..

[32] Gholami R., Ansari R., Darvizeh A., Sahmani S. (2014). Axial buckling and dynamic stability of functionally graded microshells based on the modified couple stress theory. Int. J. Struct. Stab. Dyn..

[33] Sahmani S., Ansari R., Gholami R., Darvizeh A. (2013). Dynamic stability analysis of functionally graded higher-order shear deformable microshells based on the modified couple stress elasticity theory. Compos. Part B.

[34] Pham Q.H., Nguyen P.C. (2022). Dynamic stability analysis of porous functionally graded microplates using a refined isogeometric approach. Compos. Struct..

[35] Wu C.P., Hsu C.H. (2022). Based on the consistent couple stress theory, a three-dimensional weak formulation for stress, deformation, and free vibration analyses of functionally graded microscale plates. Compos. Struct..

[36] Wu C.P., Lu Y.A. (2023). A Hermite-family *C*^1^ finite layer method for the three-dimensional free vibration analysis of exponentially graded piezoelectric microplates based on the consistent couple stress theory. Int. J. Struct. Stab. Dyn..

[37] Soldatos K.P., Ye J.Q. (1994). Three-dimensional static, dynamic, thermoelastic and buckling analysis of homogeneous and laminated composite cylinders. Compos. Struct..

[38] Ye J.Q., Soldatos K.P. (1995). Three-dimensional buckling analysis of laminated composite hollow cylinders and cylindrical panels. Int. J. Solids Struct..

[39] Leissa A.W., Turvey G.J., Marshall I.H. (1995). Buckling and postbuckling theory for laminated composite plates. Buckling and Postbuckling of Composite Plates.

[40] Soldatos K.P., Hadjigeorgiou V.P. (1990). Three-dimensional solution of the free vibration problem of homogeneous isotropic cylindrical shells and panels. J. Sound Vibr..

[41] Wu C.P., Tsai T.C. (2012). Exact solutions of functionally graded piezoelectric material sandwich cylinders by a modified Pagano method. Appl. Math. Modell..

[42] Wu C.P., Jiang R.Y. (2014). A state space differential reproducing kernel method for the buckling analysis of carbon nanotube-reinforced composite circular hollow cylinders. CMES—Comput. Model. Eng. Sci..

[43] Saada A.S. (1974). Elasticity: Theory and Applications.

[44] Vodenitcharova T., Ansourian P. (1996). Buckling of circular cylindrical shells subject to uniform lateral pressure. Eng. Struct..

[45] Shen H.S. (2003). Postbuckling analysis of pressure-loaded functionally graded cylindrical shells in thermal environments. Eng. Struct..

[46] Sofiyev A.H. (2007). Vibration and stability of composite cylindrical shells containing an FG layer subjected to various loads. Struct. Eng. Mech..

[47] Khazaeinejad P., Najafizadeh M.M., Jenabi J. (2010). On the buckling of functionally graded cylindrical shells under combined external pressure and axial compression. J. Press. Vessel Technol..

[48] Mehralian F., Beni Y.T., Ansari R. (2016). Size dependent buckling analysis of functionally graded piezoelectric cylindrical nanoshell. Compos. Struct..

[49] Mehralian F., Beni Y.T. (2017). Thermo-electro-mechanical buckling analysis of cylindrical nanoshell on the basis of modified couple stress theory. J. Mech. Sci. Technol..

[50] Kim S.E., Kim C.S. (2002). Buckling strength of the cylindrical shell and tank subjected to axially compressive loads. Thin-Walled Struct..

[51] Ng T.Y., Lam K.Y., Reddy J.N. (1998). Parametric resonance of a rotating cylindrical shell subjected to periodic axial loads. J. Sound Vibr..

